# Tyrosine 136 phosphorylation of α-synuclein aggregates in the Lewy body dementia brain: involvement of serine 129 phosphorylation by casein kinase 2

**DOI:** 10.1186/s40478-021-01281-9

**Published:** 2021-11-12

**Authors:** Kazunori Sano, Yasushi Iwasaki, Yuta Yamashita, Keiichi Irie, Masato Hosokawa, Katsuya Satoh, Kenichi Mishima

**Affiliations:** 1 Department of Physiology and Pharmacology, Faculty of Pharmaceutical Sciences, Fukuoka University, 8-19-1 Nanakuma, Jonan-ku, Fukuoka, 814-0180 Japan; 2 Department of Neuropathology, Institute for Medical Science of Aging, Aichi Medical University, Aichi, 480-1195 Japan; 3 Department of Immunological and Molecular Pharmacology, Faculty of Pharmaceutical Sciences, Fukuoka University, Fukuoka, 814-0180 Japan; 4 Department of Health Sciences, Unit of Medical and Dental Sciences, Nagasaki University Graduate School of Biomedical Sciences, Nagasaki, 852-8523 Japan

**Keywords:** α-Synuclein, Lewy body dementia, Y136 phosphorylation, S129 phosphorylation, Casein kinase 2

## Abstract

Serine 129 (S129) phosphorylation of α-synuclein (αSyn) is a central feature of Lewy body (LB) disease pathology. Although the neighboring tyrosine residues Y125, Y133, and Y136 are also phosphorylation sites, little is known regarding potential roles of phosphorylation cross-talk between these sites and its involvement in the pathogenesis of LB disease. Here, we found that αSyn aggregates are predominantly phosphorylated at Y136 in the Lewy body dementia brain, which is mediated by unexpected kinase activity of Casein kinase 2 (CK2). Aggregate formation with S129 and Y136 phosphorylation of recombinant αSyn (r-αSyn) were induced by CK2 but abolished by replacement of S129 with alanine (S129A) in vitro. Mutation of Y136 to alanine (Y136A) promoted aggregate formation and S129 phosphorylation of r-αSyn by CK2 in vitro. Introduction of Y136A r-αSyn oligomers into cultured cells exhibited increased levels of aggregates with S129 phosphorylation compared to wild-type r-αSyn oligomers. In addition, aggregate formation with S129 phosphorylation induced by introduction of wild-type r-αSyn oligomers was significantly attenuated by CK2 inhibition, which resulted in an unexpected increase in Y136 phosphorylation in cultured cells. Our findings suggest the involvement of CK2-related αSyn Y136 phosphorylation in the pathogenesis of LB disease and its potential as a therapeutic target.

**Supplementary Information:**

The online version contains supplementary material available at 10.1186/s40478-021-01281-9.

## Introduction

Lewy body (LB) diseases, including Lewy body dementia (LBD) and Parkinson’s disease (PD), are progressive diseases characterized by extensive accumulation of intracellular proteinaceous inclusions composed mainly of aggregated α-synuclein (αSyn) in the brain called LB. Although the pathological mechanisms of LB disease have not been fully elucidated, accumulation of LB, i.e., αSyn aggregates, is thought to play a significant role.

A significant proportion of αSyn accumulated within LB is phosphorylated on the C-terminal serine 129 (S129), while only a small fraction of αSyn is constitutively phosphorylated at this residue in the brain without LB pathology. Earlier in vitro and *vivo* studies yielded contrasting results regarding the significance of S129 phosphorylation (pS129) for LB formation, showing facilitatory [10, 32], inhibitory [6, 24], or no effect [17, 30] of phosphorylation on αSyn aggregation. In mice inoculated with recombinant αSyn (r-αSyn) fibrils, the S129-phosphorylated and nonphosphorylated forms were shown to cause prion-like seeding and accumulation of endogenous αSyn in the brain, and the phosphorylated form showed greater potency to induce the pathology than the nonphosphorylated form [14]. Therefore, although pS129 is not necessarily required for LB formation, it appears to induce more severe pathology.

**Fig. 1:**
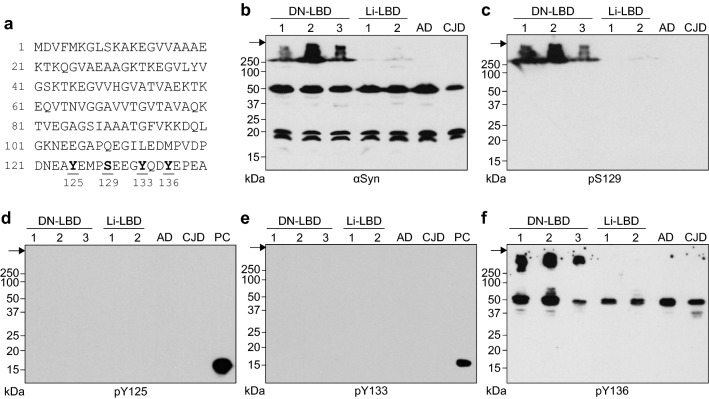
Insoluble αSyn was phosphorylated at Tyr136 as well as Ser129 in the DN-LBD brain. **a** Amino acid sequence of human αSyn. C-terminal phosphorylation sites are shown in bold and are underlined. **b**–**f** Brain lysates from DN-LBD (cases #1, #2, and #3), Li-LBD (cases #1 and #2), AD, and CJD patients were analyzed by SDS-PAGE followed by immunoblotting with **b** anti-αSyn antibody D119, **c** anti-pS129-αSyn antibody ab51253, **d** anti-pY125-αSyn antibody, **e** anti-pY133-αSyn antibody, and **f** anti-pY136-αSyn antibody. Molecular mass markers are indicated in kDa on the left side of each panel. Arrows indicate the top of the gel. In d and e, WT r-αSyn (40 μg) was incubated in the presence of 100 ng/mL Syk (S52-10G; SignalChem Pharmaceuticals) and 200 μM ATP in 100 μL of reaction buffer (20 mM Tris–HCl, pH 7.5, 50 mM KCl, and 10 mM MgCl_2_) at 37 °C for 1 h, and the sample containing WT r-αSyn (2 μg) was loaded as a positive control (PC)

αSyn tyrosines Y125, Y133, and Y136 are located in close proximity to S129 at the C-terminus (Fig. 1a), which raises questions regarding whether these tyrosine residues are phosphorylated and whether there are interactions among the phosphorylated residues, including S129 and its association with αSyn aggregate formation. Human r-αSyn incubated with spleen tyrosine kinase (Syk) [22] and αSyn in pervanadate-treated cultured human cells [8] have been reported to be phosphorylated at Y125, Y133, and Y136. Several studies have demonstrated the presence of αSyn phosphorylated at Y125 (pY125) in the human brain. An immunohistochemical study identified pY125 within LB in a case of familial PD with G51D mutation [15]. Immunoblotting analysis detected pY125 at similar levels in PD and control brains [19] but at higher levels in control than LBD brains [7]. Other studies showed that pY125 is not a component of LB in LBD and PD by immunohistochemical and immunoblotting analyses [9] and mass spectrometry [1]. pY125 has been reported to inhibit toxic oligomer formation of αSyn in *Drosophila* [7], whereas an in vitro study showed that pY125 had no influence on aggregation of synthetic αSyn [11, 30]. Therefore, αSyn appears to be phosphorylated at Y125 in the human brain, but the role of pY125 in αSyn aggregate formation has not been fully elucidated. Immunoblotting analysis indicated the presence of phosphorylated Y133 (pY133) at similar levels in LBD, PD, and control brains [9], suggesting that pY133 may not be crucial for LB pathology. In contrast, there have been few studies regarding the presence of phosphorylated Y136 (pY136) in the human brain and its physiological roles and implications in the pathogenesis of LB disease.

Casein kinase (CK) 1 and 2 are ubiquitous serine/threonine protein kinases expressed in all eukaryotes. Although both CK1 and CK2 have been shown to constitutively phosphorylate S129 of αSyn in vitro [23, 35], CK2 rather than CK1 was demonstrated to be the major enzyme in the brain that phosphorylates S129 of human αSyn [13]. In a study using transgenic mice expressing human αSyn, S129-phosphorylated αSyn was shown to be preferentially colocalized with CK2 rather than CK1 in the nuclei [34]. Furthermore, the catalytic (α) and regulatory (β) subunits of CK2 have been shown to be present in the insoluble fraction of the LBD brain [13] and in LB in the PD brain [26], respectively. In contrast, CK1 isoform delta (CK1δ) was shown to be absent in LB in the PD and LBD brain [31]. These studies suggested a role of CK2 in LB formation. CK2 has been shown to exhibit tyrosine kinase activity in cultured mammalian cells [3, 33]. In yeast, CK2 has also been reported to phosphorylate tyrosine 184 (Y184) of the nucleolar immunophilin, Fpr3, through prior phosphorylation at serine 186 (S186) at the + 2 position [37], suggesting that phosphorylated serine residues play a key role in mediating phosphorylation of neighboring tyrosine residues. In the case of αSyn, pS129 and pY125 were shown to have no effect on each other in *Drosophila* by immunoblotting analysis [7] and in vitro by immunoblotting and MALDI-TOF mass spectrometry (MS) [11], while a study using in vitro NMR spectroscopy reported that pY125 primes efficient phosphorylation of S129 by CK1 [16]. Therefore, although tyrosines Y125, Y133, and Y136 are located in close proximity to S129 at the C-terminus of αSyn, it is not clear whether there is phosphorylation cross-talk between these sites, and whether such cross-talk is functionally relevant to the pathogenesis of LB disease. Here, we report that insoluble αSyn is highly phosphorylated at Y136 as well as S129 in the LBD brain. Furthermore, experimental manipulation of the phosphorylation state demonstrated that phosphorylation of S129 by CK2 mediates phosphorylation of Y136 and thus suppresses the formation of αSyn aggregates.

## Materials and methods

### Reagents

Anti-αSyn polyclonal antibody (D119) and monoclonal antibody (Syn204) were obtained from Bioworld Technology Inc. and Cell Signaling Technology, respectively, and were used for western blotting. Anti-pS129-αSyn polyclonal antibody (D1R1R) and anti-β-actin polyclonal antibody (ab8227) were obtained from Cell Signaling Technology and Abcam, respectively, and were used for western blotting. Monoclonal antibody specific for pS129-αSyn (ab51253) and polyclonal antibodies against pY125-αSyn (ab10789), pY133-αSyn (ab194910), and pY136-αSyn (ab194775) used for western blotting and immunohistochemical staining were purchased from Abcam. Casein kinase 2 (CK2, P6010L) was obtained from New England Biolabs Inc. ATP (A2383) was acquired from Sigma-Aldrich.

### Brains of patients

LBD brain tissues were obtained at autopsy from five patients with histopathologically confirmed clinical diagnosis. Three of these subjects had diffuse neocortical LBD (DN-LBD), and the remaining two cases had limbic LBD (Li-LBD) according to Braak staging. Brain tissues were obtained at autopsy from a patient with a neuropathological diagnosis of Alzheimer’s disease (AD) based on the presence of neurofibrillary tangles and neuritic plaque. The brain specimens showed pure AD with little or no coexisting LB disease. Prion disease brain tissue specimens were obtained at autopsy from a patient with sporadic Creutzfeldt–Jakob disease (CJD), which was diagnosed as the classical MM1 subtype according to the genotype at codon 129 of the *PRNP* gene and the physicochemical properties of abnormal prion protein (PrP^Sc^). For DN-LBD cases #1, #2, and #3, the individuals were 92, 80, and 91 years old at the time of death, respectively. Li-LBD cases #1 and #2 were 80 and 87 years old at the time of death, respectively. The patients with AD and CJD were 82 and 75 years old at the time of death, respectively. All tissues were taken from the frontal cortex and stored at − 80 °C.

### Preparation of brain lysates

Brain tissues were lysed with Triton-deoxycholate (DOC) lysis buffer (50 mM Tris–HCl, pH 7.5, containing 150 mM NaCl, 0.5% Triton X-100, 0.5% sodium deoxycholate, 2 mM EDTA, and protease inhibitors) for 30 min at 4 °C. After centrifugation for 2 min at 2000 × *g*, the supernatant was collected and stored at − 80 °C until use. The total protein concentration of the lysates was measured using a bicinchoninic acid (BCA) protein assay kit (23227; Pierce).

### Recombinant human α-synuclein expression and purification

The purification of recombinant human αSyn (r-αSyn) and mutants was performed as described previously [29]. Briefly, plasmids carrying the DNA sequence encoding N-terminal His-tagged human wild-type (WT) or nonphosphorylatable mutants with substitution of alanine for S129 (S129A) or Y136 (Y136A) were subcloned into the vector pET11a (69436-3; Novagen). The products encoded on the plasmid were overexpressed in competent BL21 DE3 *Escherichia coli* cells (DS250; BioDynamics Laboratory) at 37 °C for 16 h using MagicMedia *E. coli* Expression Medium (K6815; Invitrogen). The bacterial pellets were suspended in CelLytic B (B7435; Sigma-Aldrich) in the presence of 1 g/mL lysozyme (120-02674; Wako) and 500 U/mL benzonase nuclease (70664-3; Novagen). The lysate was centrifuged at 10,000 rpm for 30 min at 4 °C, and the supernatant was incubated with Ni–NTA Superflow resin (30430; Qiagen) at room temperature for 30 min, and then loaded onto a gravity flow column (Muromac mini-column; Muromachi Chemical Inc.). The His-tagged proteins were eluted with a buffer containing 300 mM NaCl, 50 mM Tris–HCl (pH 8.0), and 250 mM imidazole, and dialyzed against 10 mM phosphate buffer (pH 7.0) in cellulose dialysis tubing (68035; Thermo Scientific) at 4 °C overnight. Cleavage of the His-tagged from the proteins followed by removal of uncleaved His-tagged proteins was performed using the TAGZyme system (34300; Qiagen). The non-tagged proteins were then dialyzed against deionized distilled water in cellulose dialysis tubing at 4 °C overnight and filtered with a 0.2-µm syringe filter (SLLGH25; Millipore). The purity of r-αSyn was ≥ 99.9% as estimated by SDS-PAGE and western blotting. After purification, aliquots of r-αSyn were stored at − 80 °C until use.

### In vitro phosphorylation of recombinant α-synuclein

r-αSyn (40 μg) was incubated in the presence of 400 units of casein kinase 2 and 200 μM ATP in 100 μL of reaction buffer (20 mM Tris–HCl, pH 7.5, 50 mM KCl, and 10 mM MgCl_2_) at 37 °C.

### Gel electrophoresis and immunoblotting

For SDS-PAGE, samples were boiled for 5 min at 95 °C with SDS loading buffer (62.5 mM Tris–HCl, pH 6.8, containing 5% 2-mercaptoethanol, 2% SDS, 5% sucrose, and 0.005% bromophenol blue) and were separated by 15% SDS-PAGE. For BN-PAGE, samples were prepared in a buffer containing 0.25% Coomassie Brilliant Blue G-250 and separated by 4–16% Bis–Tris native PAGE (BN1004BOX; Invitrogen). The proteins were transferred onto Immobilon-P membranes (IPVH304F0; Millipore) in transfer buffer containing 15% methanol followed by blocking with 5% nonfat dry milk in TBST (10 mM Tris–HCl, pH 7.8, 100 mM NaCl, 0.1% Tween 20) for 2 h at 4 °C. Membranes were then probed with specific primary antibodies (1:2500) and appropriate horseradish peroxidase-conjugated secondary antibody (111-035-003 or 115-035-003, 1:5000; Jackson ImmunoResearch Labs). Immunoreactive bands were visualized using Chemi-Lumi One L (07880-70; Nacalai Tesque) or ECL prime Western Blotting Detection Reagents (PRN2232; GE Healthcare Life Sciences).

### Immunohistochemical staining

The brain tissues were fixed in 20% neutral buffered formalin, embedded in paraffin, cut into Sects. 8 μm thick with a microtome, and placed on glass slides. After deparaffinization and rehydration, the tissue sections were heated for 40 min at 98 °C for antigen retrieval. All sections were immersed in hydrogen peroxide (0.3%) solution for 10 min to quench endogenous peroxidase activity and incubated with specific primary antibodies (1:100) diluted in PBS containing 1% BSA for 2 h. Primary antibody binding was detected by the labeled streptavidin–biotin method (DAKO). Peroxidase-conjugated streptavidin was visualized with 3′3-diaminobenzidine (7411-49-6; Wako) as the chromogen. Immunostained sections were lightly counterstained with Mayer’s hematoxylin.

### Gel staining and in-gel digestion

After separation of proteins by SDS-PAGE, the gels were stained at room temperature for 2 h with Coomassie Brilliant Blue solution (11642; Nacalai Tesque). The stained bands near 16 kDa were excised and soaked in 50 mM Tris–HCl, pH 8.0, containing 50% acetonitrile for 30 min. The gel was dried in a Speed-Vac (Savant) and incubated in 50 mM triethylammonium bicarbonate containing proteomics grade trypsin (T7575; Sigma-Aldrich) at 37 °C for 20 h. The digests were extracted from the gel with 100–200 μL of 0.1% TFA containing 60% acetonitrile. These extracts were evaporated in a Speed-Vac and stored at − 80 °C until assayed.

### Nano-flow liquid chromatography-ion trap mass spectrometry (LC–MS/MS)

Peptides were resuspended in 0.1% formic acid containing 2% acetonitrile. Measurements were performed on a nano-flow high-performance liquid chromatography (HPLC) system (EASY-nLC 1200; Thermo Fisher Scientific). The samples were loaded onto packed nano-capillary columns (0.075 mm I.D. × 125 mm L, particle diameter 3 μm, NTCC-360/75-3-123; Nikkyo Technos Co., Ltd.), which were eluted at a flow rate of 300 nL/min with a 2–80% linear gradient of acetonitrile for 80 min. Eluting peptides were detected with an ion trap mass spectrometer (QExactive HF; Thermo Fisher Scientific). For ionization, the spray voltage and capillary temperature were set to 2.0 kV and 250 °C, respectively. The mass acquisition method consisted of one full MS survey scan with an Orbitrap resolution of 60,000 followed by mass spectrometry (MS/MS) of the most abundant precursor ions from the survey scan with an Orbitrap resolution of 15,000. Dynamic exclusion for MS/MS was set to 30 s. MS was performed with a scan range of 350–1800 m/z in positive ion mode, followed by data-dependent MS/MS using the HCD operating mode on the top 15 ions in order of abundance. The data were analyzed with Proteome Discoverer (Thermo Fisher Scientific) and Mascot software (Matrix Science).

### Transmission electron microscopy (TEM)

Negative staining was performed on 400 mesh copper grids with a carbon support film. Aliquots of the samples were adsorbed onto the grids, and the residual solution was carefully removed from the grid surface using filter paper. The grids were stained with 2% uranyl acetate. Once dry, the samples were viewed with a transmission electron microscope (TEM) (JEM-2000FX; JEOL) at 200 kV.

### Thioflavin T (ThT) assay

r-αSyn (40 μg) was prepared in 96-well optical black-bottomed plates (265301; Nunc) in 100 μL of reaction buffer (20 mM Tris–HCl, pH 7.5, 50 mM KCl, 10 mM MgCl_2_, and 10 μM thioflavin T [ThT]). The 96-well plates were covered with sealing tape (236366; Nunc) and incubated at 40 °C in a plate reader (FLUOstar Omega plate reader; BMG Labtech) with intermittent shaking, consisting of 30 s of double orbital shaking at 500 rpm and no shaking for 30 s. ThT fluorescence intensity on the bottom of the plates was measured every 10 min to monitor the kinetics of amyloid fibril formation using monochromators with excitation and emission wavelengths of 450 ± 10 and 480 ± 10 nm, respectively. Lag phase was defined as the time required to reach fluorescence greater than or equal to the mean fluorescence intensity for all samples within the first 24 h of reaction plus 4 × the standard deviation.

### Preparation of r-αSyn seeds

r-αSyn (40 μg) was incubated in 20 mM Tris–HCl, pH 7.5, containing 50 mM KCl, and 10 mM MgCl_2_, at 37 °C under agitation at 2000 rpm for 3 days. As a control for agitated r-αSyn, the mix was prepared without incubation and agitation immediately before use. The morphology of r-αSyn seeds was confirmed by TEM.

### Cell culture, treatment of r-αSyn seeds, and transfection of plasmids

Human neuroblastoma SH-SY5Y cells were maintained at 37 °C in 5% CO_2_ in DMEM/Ham’s F12 medium (042-30795; Wako) containing 15% fetal bovine serum (SH30910; GE Healthcare Hyclone), penicillin–streptomycin (168-23191; Wako), and MEM Non-essential Amino Acids Solution (139-15651; Wako). Introduction of r-αSyn seeds and/or pcDNA3.1 plasmid encoding human WT or Y136A mutant αSyn into cells was performed using Lipofectamine™ LTX (Invitrogen) according to the manufacturer’s instructions. Lipofectamine/r-αSyn seeds and/or plasmid complexes were prepared in Optimem (31985062; Gibco) by mixing Lipofectamine LTX reagent in the presence or absence of r-αSyn seeds (4 μg) or plasmid (5 μg). Cells were cultured to 40–50% confluence in 6-well plates and treated with the complexes. For CK2 inhibitor treatment, cells were incubated with 10 or 100 nM 4,5,6,7-tetrabromobenzotriazole (TBB, ab120988; Abcam) for 1 h before addition of the complexes. cells were incubated for 3 d after introduction of r-αSyn seeds and/or plasmid.

### Preparation of lysate from cell culture

Cells were washed with PBS and harvested, and cellular proteins were extracted with Triton-DOC lysis buffer. The total protein concentration of the lysates was measured using a BCA protein assay kit (23227; Pierce).

### Statistics

Statistical analyses were performed with OriginPro 2015 (OriginLab). The 2-tailed Student’s *t* test was used for comparisons between two groups. One-way analysis of variance (ANOVA) followed by the Tukey–Kramer test was used for comparisons among more than two groups. In all analyses, *P* < 0.05 was taken to indicate statistical significance.

## Results

### Insoluble aggregates of αSyn are predominantly phosphorylated on Y136 as well as S129 within the C-terminal region in the LBD brain

Brain lysates from LBD patients were analyzed by SDS-PAGE followed by immunoblotting with anti-αSyn antibody, and all exhibited a band at approximately 20 kDa with another band just below 20 kDa likely corresponding to full-length and cleaved αSyn, respectively (Fig. 1b). All lysates also exhibited an αSyn-positive band at approximately 50 kDa, which was presumably due to the oligomeric and/or ubiquitinated αSyn. Although these αSyn-positive bands did not differ significantly between cases, multimeric αSyn with molecular weight > 250 kDa was observed only in three cases of DN-LBD (Fig. 1b, Additional file 1: Fig. S1). Immunostaining with an antibody against pS129-αSyn detected only a major band at > 250 kDa in all cases of DN-LBD (Fig. 1c, Additional file 1: Fig. S1). A band of > 250 kDa was detected with antibodies against αSyn and pS129-αSyn in Li-LBD case #2, but not case #1, but the intensity was significantly lower than in DN-LBD (Fig. 1b, c), indicating that multimer formation and pS129 of αSyn are relevant to disease progression. Consistent with our previous report [29], these results suggest that the detergent-insoluble multimer of αSyn highly phosphorylated at S129 with molecular weight > 250 kDa is present in the DN-LBD brain. We next examined the presence of αSyn phosphorylated at C-terminal tyrosine residues surrounding S129. No signals were detected with antibodies against pY125-αSyn and pY133-αSyn in any cases (Fig. 1d, e). A 50-kDa band was detected with an antibody against pY136-αSyn in all cases (Fig. 1f, Additional file 1: Fig. S1). Moreover, the three cases of DN-LBD showed a strong immunoreactive band with mass > 250 kDa (Fig. 1f, Additional file 1: Fig. S1). These results suggest that the insoluble aggregates of αSyn are predominantly phosphorylated on Y136 as well as S129 within the C-terminal region in the LBD brain.

**Fig. 2:**
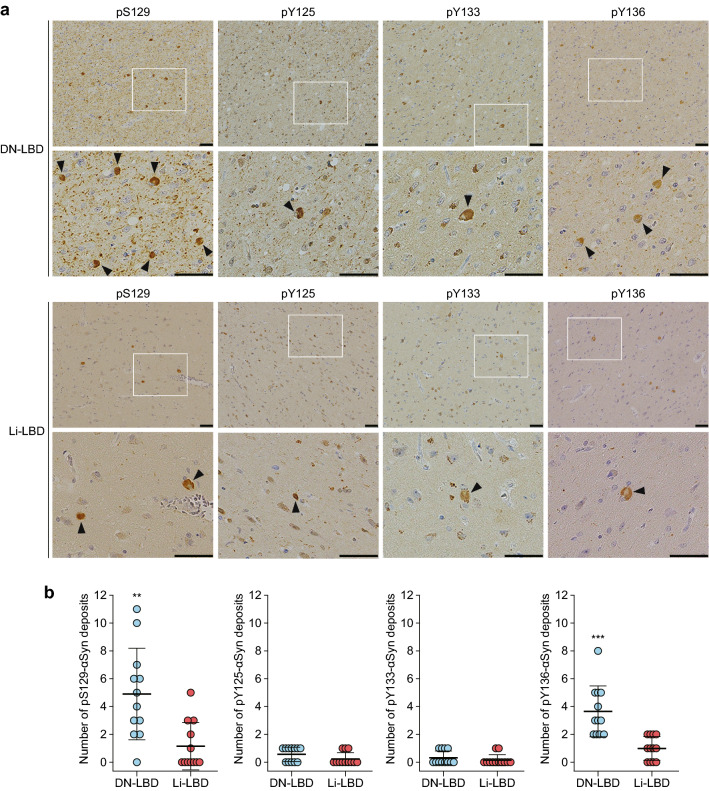
Deposits of phosphorylated αSyn at C-terminal tyrosine residues are present in LBD brain. **a** Immunohistochemical staining using antibodies against pS129-αSyn ab51253, pY125-αSyn, pY133-αSyn, and pY136-αSyn in the frontal cortex from two patients with DN-LBD (case #3) and Li-LBD (case #2). Regions surrounded by white rectangles in the upper panels are magnified and shown in the lower panels. Typical pathological αSyn deposits are indicated by arrowheads. Scale bars, 50 μm. **b** The number of phosphorylated αSyn deposits > 2 μm^2^ was counted in 12 randomly selected areas of 10 mm^2^ in each tissue specimen using ImageJ. Data are presented as means ± standard deviation. Statistical significance was determined using the 2-tailed Student’s *t* test. **s < 0.01, ***P < 0.001 vs. Li-LBD

We next examined phosphorylation of the C-terminus of αSyn in sections of the frontal cortex of DN-LBD and Li-LBD brains by immunohistochemical analysis. Consistent with our previous study [29], the deposits showed positive staining for pS129-αSyn, and were also positive for pY136-αSyn in both DN-LBD and Li-LBD brains (Fig. 2a). The numbers of pS129-αSyn and pY136-αSyn deposits were significantly greater in DN-LBD than Li-LBD (Fig. 2b). Deposits were not detected in the brain of a non-DLB patient by immunohistochemical analysis using anti-pS129-αSyn antibody (Additional file 1: Fig. S2). Both DN-LBD and Li-LBD brains showed positive staining with anti-pY125-αSyn and anti-pY133-αSyn antibodies (Fig. 2a), but pT125-αSyn and pY133-αSyn deposits were present at low levels in both DN-LBD and Li-LBD compared to pS129-αSyn and pY136-αSyn deposits in DN-LBD (Fig. 2b). The lack of detectable pY125 and pY133 in the brain by immunoblotting was likely due to low levels of the deposits, and phosphorylated αSyn may not be detected by immunoblotting in brains with less than two phosphorylated deposits per 10 mm^2^ of tissue. There were no significant differences in the numbers of pY125-αSyn and pY133-αSyn deposits between the two types of LBD (Fig. 2b). Thus, the results of immunohistochemical analysis indicated the presence of pS129-αSyn- and pY136-αSyn-positive deposits in larger amounts in the brains of patients with DN-LBD than Li-LBD, and that pY125-αSyn and pY133-αSyn are present in relatively small amounts in the brains of patients with both types of LBD.

### Ser129 phosphorylation by CK2 promotes the formation of insoluble αSyn aggregates in vitro

**Fig. 3:**
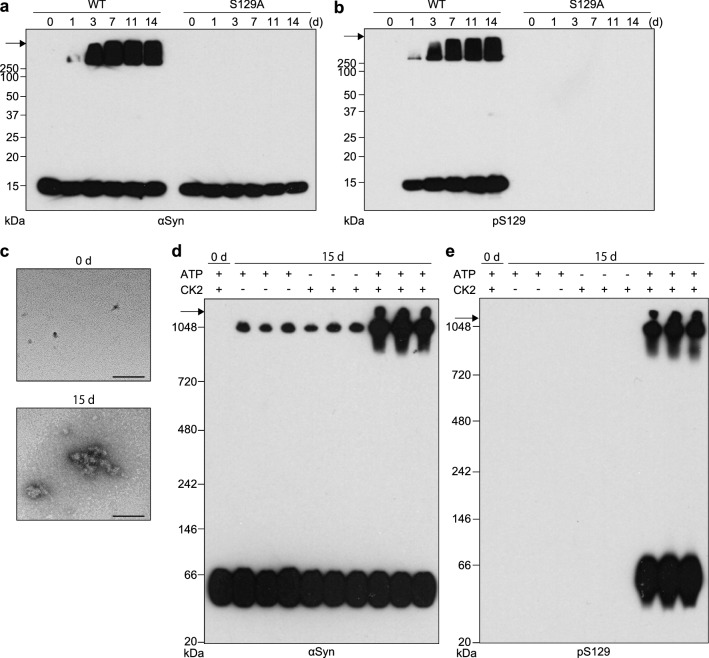
Ser129 phosphorylation by CK2 accelerates αSyn aggregate formation. **a**, **b** WT r-αSyn or S129A r-αSyn after 0–14 days of incubation with CK2 and ATP was analyzed by SDS-PAGE followed by immunoblotting with **a** anti-αSyn antibody D119 and **b** anti-pS129-αSyn antibody ab51253. **c** WT r-αSyn after 0 or 15 days of incubation with CK2 and ATP was examined by TEM. Bars, 100 nm. **d**, **e** WT r-αSyn after 0 or 15 days of incubation in the presence (+) or absence (−) of CK2 or ATP was analyzed by BN-PAGE followed by immunoblotting with **d** anti-αSyn antibody D119 and **e** anti-pS129-αSyn antibody ab51253. Molecular mass markers are indicated in kDa on the left side of each panel. Arrows indicate the top of the gel. In **a**, **b**, **d** and **e**, one representative blot from three independent experiments is shown

Analysis of WT r-αSyn by SDS-PAGE followed by immunoblotting with anti-αSyn antibody revealed detergent-insoluble aggregates > 250 kDa after 15 days of incubation, which were formed more efficiently in the presence of both CK2 and ATP than in the absence of either (Additional file 1: Fig. S3). Insoluble aggregates and 16-kDa WT r-αSyn monomer incubated with CK2 and ATP were detected by anti-pS129-αSyn antibody, whereas no immunoreactivity was detected with anti-pS129-αSyn antibody for WT r-αSyn incubated under other conditions (Additional file 1: Fig. S3). The insoluble aggregates of S129-phosphorylated WT r-αSyn were detected after 1 day of incubation (Fig. 3a, b). Meanwhile, S129A r-αSyn incubated with CK2 and ATP was not phosphorylated at Ser129 and formed no insoluble aggregates (Fig. 3a, b). TEM revealed that WT r-αSyn subjected to 15-day incubation with CK2 and ATP consisted exclusively of amorphous aggregates, but no such aggregates were seen without incubation (Fig. 3c). The amorphous aggregates were present in relatively small amounts in WT r-αSyn incubated in the absence of CK2 and ATP (Additional file 1: Fig. S4). These results were consistent with a previous report [29] indicating that CK2-induced pS129 accelerated aggregate formation of WT r-αSyn, and the insoluble aggregates were observed in the mass range > 250 kDa as in the DN-LBD brain. Blue native (BN)-PAGE followed by immunoblotting analysis with anti-αSyn antibody of WT r-αSyn incubated for 15 days revealed the formation of αSyn aggregates with a molecular weight of ~ 1048 kDa in addition to a band of monomeric αSyn at approximately 60 kDa (Fig. 3d). The aggregated WT r-αSyn was also formed more efficiently in the presence of both CK2 and ATP than in the absence of either, suggesting that pS129 accelerates aggregate formation of αSyn (Fig. 3d, e).

### CK2 phosphorylates Y125 and Y136 of aggregated αSyn through prior S129 phosphorylation in vitro

**Fig. 4:**
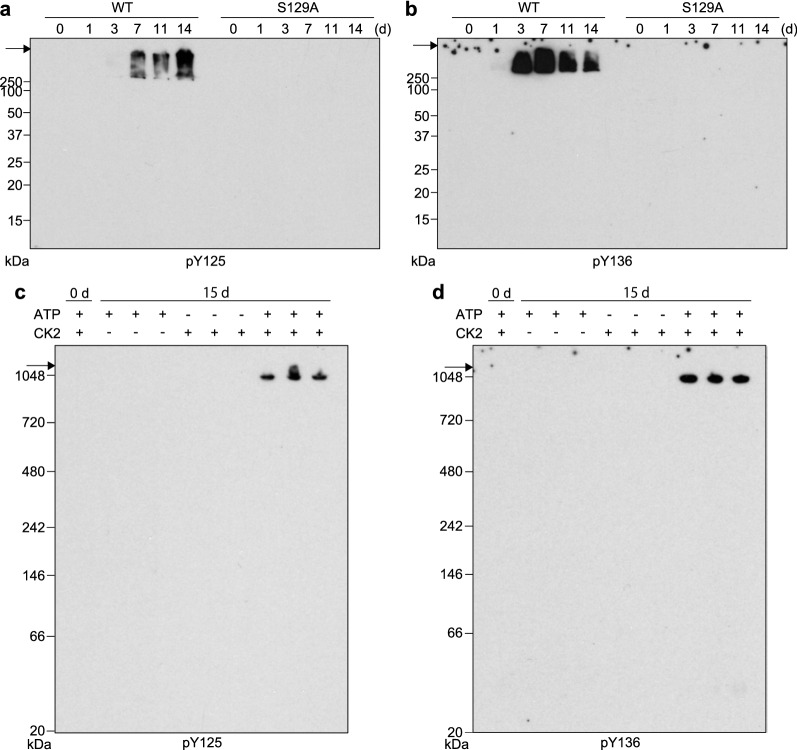
Ser129 phosphorylation by CK2 elicits Tyr125 and Tyr136 phosphorylation of αSyn. **a**, **b** WT r-αSyn or S129A r-αSyn after 0–14 days of incubation with CK2 and ATP was analyzed by SDS-PAGE followed by immunoblotting with **a** anti-pY125-αSyn antibody and **b** anti-pY136-αSyn antibody. **c**, **d** WT r-αSyn after 0 or 15 days of incubation in the presence (+) or absence (−) of CK2 or ATP was analyzed by BN-PAGE followed by immunoblotting with **c** anti-pY125-αSyn antibody and **d** anti-pY136-αSyn antibody. Molecular mass markers are indicated in kDa on the left side of each panel. Arrows indicate the top of the gel. In **a**–**d**, one representative blot from three independent experiments is shown

WT r-αSyn or S129A r-αSyn was incubated with CK2 and ATP for 7 days, separated by SDS-PAGE, and the gel portion corresponding to a molecular weight of ~ 16 kDa was cut out as shown in Additional file 1: Fig. S5. The gel slice was digested with trypsin and analyzed by LC–MS/MS. As shown in Table 1, we identified 23 and seven phosphorylated peptides from WT and S129A r-αSyn, respectively. LC–MS/MS analysis detected pS129 in WT r-αSyn. Both WT r-αSyn and S129A r-αSyn contained pS87. WT r-αSyn showed phosphorylation at four threonine residues, i.e., T44, T54, T59, and T64, while S129A r-αSyn showed phosphorylation at five threonine residues, i.e., T22, T44, T54, T59, and T75. There have been no previous reports of phosphorylation of αSyn at T22, T44, T54, T59, T64, T75, and S87 by CK2, and the number of peptides including these residues with phosphorylation was very low in comparison to peptides with pS129 (Table 1). Therefore, CK2 may phosphorylate r-αSyn on these residues at very low levels in vitro. Moreover, peptides with pY125 and pY136 were identified in WT r-αSyn. The number of peptides with pY136 was comparable to that of peptides with pS129, and only one peptide with pY125 was detected. In contrast, no peptides with C-terminal tyrosine phosphorylation were identified in S129A r-αSyn. After SDS-PAGE of WT r-αSyn, immunoblotting with anti-pY125-αSyn antibody and anti-pY136-αSyn antibody detected detergent-insoluble aggregates > 250 kDa after 7 and 3 days of incubation with CK2 and ATP, respectively (Fig. 4a, b). No immunoreactivity was detected on blots of S129A with anti-pY125-αSyn or anti-pY136-αSyn antibody (Fig. 4a, b). In BN-PAGE followed by immunoblotting analysis of WT r-αSyn incubated for 15 days, bands of approximately 1048 kDa were detected with antibodies against pY125-αSyn and pY136-αSyn only in the presence of CK2 and ATP (Fig. 4c, d). Moreover, pS129 and pY136 were abolished and the formation of insoluble aggregates was significantly inhibited by the addition of alkaline phosphatase in WT r-αSyn incubated with CK2 and ATP (Additional file 1: Fig. S6). These results suggested that CK2 phosphorylates Y125 and Y136 of aggregated WT r-αSyn in vitro, and that the tyrosine phosphorylation is mediated through pS129.

**Table 1:** List of identified peptides derived from r-αSyn incubated with CK2 and ATP for 7 days

Query	Start	End	Observed	Mr(expt)	Mr(calc)	ppm	Score	Expect	Peptide
*WT*									
5284	44	58	802.9034	1603.792	1603.797	− 2.97	65	3.5E−07	K.TpKEGVVHGVATVAEK.T
11012	46	80	1172.286	3513.836	3513.808	8.02	51	8.4 E−06	K.EGVVHGVATpVAEKTKEQVTN*VGGAVVTGVTAVAQK.T
8325	59	80	746.3851	2236.134	2236.147	− 5.77	47	0.000021	K.TKEQVTpNVGGAVVTGVTAVAQK.T
8336	59	80	1120.068	2238.122	2238.115	3.44	21	0.0083	K.TpKEQ*VTN*VGGAVVTGVTAVAQK.T
11170	59	96	924.9822	3695.9	3695.914	− 3.77	76	2.4 E−08	K.TpKEQVTNVGGAVVTGVTAVAQKTVEGAGSIAAATGFVK.K
5009	81	96	779.8777	1557.741	1557.744	− 1.95	88	1.6 E−09	K.TVEGAGSpIAAATGFVK.K
12198	98	140	1228.245	4908.951	4908.947	0.96	20	0.011	K.DQLGKN*EEGAPQEGILEDMPVDPDN*EAYEMPSpEEGYQDYEPEA
12214	98	140	1232.002	4923.98	4923.958	4.6	34	0.00044	K.DQLGKN*EEGAPQEGILEDMoPVDPDNEAYEMPSEEGYQDYpEPEA
12225	98	140	1235.754	4938.985	4938.969	3.36	21	0.01	K.DQLGKNEEGAPQEGILEDMoPVDPDNEAYEMoPSEEGYQDYpEPEA
12226	98	140	1235.756	4938.994	4938.969	5.24	30	0.0011	K.DQLGKNEEGAPQEGILEDMoPVDPDNEAYpEMoPSEEGYQDYEPEA
12228	98	140	1236.002	4939.979	4939.953	5.31	23	0.0073	K.DQLGKNEEGAPQEGILEDMoPVDPDN*EAYEMoPSEEGYQDYpEPEA
12229	98	140	1236.003	4939.982	4939.953	5.91	26	0.0032	K.DQ*LGKNEEGAPQEGILEDMoPVDPDNEAYEMoPSEEGYQDYpEPEA
11769	103	140	1092.433	4365.703	4365.693	2.34	21	0.0077	K.NEEGAPQEGILEDMPVDPDNEAYEMPSEEGYQDYpEPEA
11770	103	140	1456.242	4365.704	4365.693	2.61	18	0.017	K.NEEGAPQEGILEDMPVDPDNEAYEMPSpEEGYQDYEPEA
11805	103	140	1461.57	4381.688	4381.688	0.11	24	0.004	K.NEEGAPQEGILEDMPVDPDNEAYEMoPSpEEGYQDYEPEA
11806	103	140	1461.572	4381.693	4381.688	1.19	22	0.0069	K.NEEGAPQEGILEDMPVDPDNEAYEMoPSpEEGYQDYEPEA
11807	103	140	1461.572	4381.694	4381.688	1.45	28	0.0015	K.NEEGAPQEGILEDMoPVDPDNEAYEMPSpEEGYQDYEPEA
11808	103	140	1096.431	4381.696	4381.688	1.93	25	0.0034	K.NEEGAPQEGILEDMPVDPDNEAYEMoPSpEEGYQDYEPEA
11809	103	140	1461.576	4381.707	4381.688	4.54	28	0.0017	K.NEEGAPQEGILEDMPVDPDNEAYEMoPSpEEGYQDYEPEA
11851	103	140	1100.425	4397.671	4397.682	− 2.69	31	0.00073	K.NEEGAPQEGILEDMoPVDPDNEAYEMoPSpEEGYQDYEPEA
11855	103	140	1466.906	4397.696	4397.682	3.13	31	0.00086	K.NEEGAPQEGILEDMoPVDPDNEAYEMoPSEEGYQDYpEPEA
11856	103	140	1466.908	4397.701	4397.682	4.29	24	0.004	K.NEEGAPQEGILEDMoPVDPDNEAYEMoPSEEGYQDYpEPEA
11870	103	140	1467.57	4399.687	4399.651	8.37	24	0.0039	K.NEEGAPQEGILEDMoPVDPDN*EAYEMoPSpEEGYQDYEPEA
*S129A*									
3243	11	23	691.3577	1380.701	1380.701	− 0.32	28	0.0014	K.AKEGVVAAAEKTpK.Q
1528	22	32	570.2711	1138.528	1138.538	− 9.45	46	0.000028	K.TpKQGVAEAAGK.T
4891	44	58	802.8981	1603.782	1603.797	− 9.67	60	9.1 E−07	K.TpKEGVVHGVATVAEK.T
3201	46	58	688.3341	1374.654	1374.654	− 0.57	42	0.00006	K.EGVVHGVATpVAEK.T
8000	59	80	1119.079	2236.144	2236.147	− 1.15	64	4.2 E−07	K.TpKEQVTNVGGAVVTGVTAVAQK.T
8001	59	80	746.3887	2236.144	2236.147	− 1.02	25	0.003	K.TKEQVTNVGGAVVTGVTpAVAQK.T
5372	81	97	843.9226	1685.831	1685.839	− 4.98	52	6.4 E−06	K.TVEGAGSpIAAATGFVKK.D

p indicates phosphorylation sites; o indicates oxidation site

*Indicates deamidation site

### Y136 phosphorylation prevents S129 phosphorylation and aggregate formation of αSyn in vitro

**Fig. 5:**
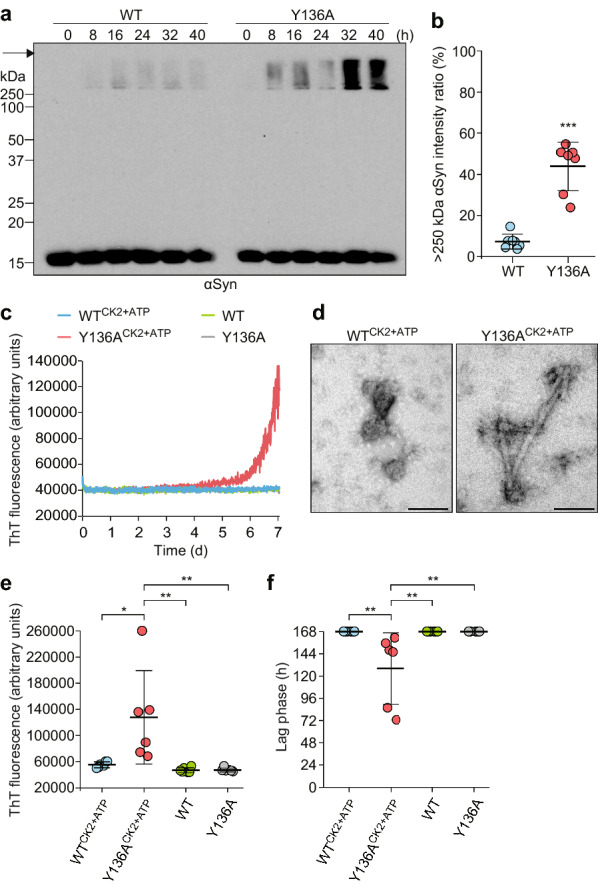
Blocking Tyr136 phosphorylation promotes aggregation and amyloid fibril formation of αSyn. **a** WT r-αSyn or Y136A r-αSyn after 0–40 h of incubation with CK2 and ATP was analyzed by SDS-PAGE followed by immunoblotting with anti-αSyn antibody D119. Molecular mass markers are indicated in kDa on the left side of each panel. Arrows indicate the top of the gel. **b** Intensity ratios (%) of immunoreactive > 250-kDa αSyn after 8 h of incubation were quantified in seven independent experiments. Data are presented as means ± standard deviation. Statistical significance was determined using the 2-tailed Student’s *t* test. ***P < 0.001 vs. WT r-αSyn. **c** ThT assays were performed in reactions with WT r-αSyn in the presence (WT^CK2+ATP^) or absence (WT) of CK2 and ATP or in reactions with Y136A r-αSyn in the presence (Y136A^CK2+ATP^) or absence (Y136A) of CK2 and ATP. The results show the kinetics of ThT fluorescence from one representative of six replicate wells for each condition. **d** The end products from reactions with WT^CK2+ATP^ or Y136A^CK2+ATP^ were examined by TEM. Bars, 100 nm. Values of **e** maximal fluorescence intensities and **f** lag phase obtained in six individual wells in ThT assay are plotted. Data are presented as means ± standard deviation. Statistical significance was determined using one-way ANOVA followed by Tukey–Kramer test. *P < 0.05, **P < 0.01

**Fig. 6:**
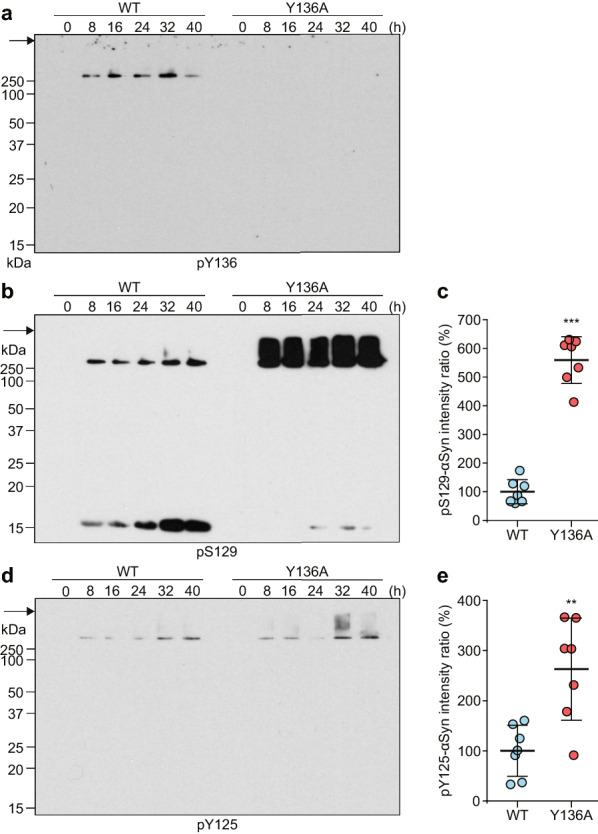
Blocking Tyr136 phosphorylation promotes phosphorylation of αSyn at Ser129 and Tyr125 induced by CK2. WT r-αSyn or Y136A r-αSyn after 0–40 hours of incubation with CK2 and ATP was analyzed by SDS-PAGE followed by immunoblotting with **a** anti-pY136-αSyn antibody, **b** anti-pS129-αSyn antibody ab51253, and **d** anti-pY125-αSyn antibody. Molecular mass markers are indicated in kDa on the left side of each panel. Arrows indicate the top of the gel. Intensity ratios (%) of immunoreactive **c** pS129-αSyn, the sum of > 250-kDa and 16-kDa forms, and **e** > 250-kDa pY125-αSyn after 8 h of incubation were quantified in seven independent experiments. Data are presented as means ± standard deviation. Statistical significance was determined using the 2-tailed Student’s *t* test. **P < 0.01, ***P < 0.001 versus WT r-αSyn

On SDS-PAGE followed by immunoblotting, Y136A r-αSyn incubated for 14 days also exhibited detergent-insoluble aggregates > 250 kDa, which were most strongly detected in the presence of both CK2 and ATP (Additional file 1: Fig. S7). Insoluble aggregates > 250 kDa as well as 16 kDa monomer of Y136A r-αSyn incubated with CK2 and ATP were detected by anti-pS129-αSyn antibody (Additional file 1: Fig. S7), while no immunoreactivity was detected with anti-pY136-αSyn antibody in any samples (Additional file 1: Fig. S7). Although no αSyn-positive band > 250 kDa of WT r-αSyn or Y136A r-αSyn was observed within 40 h of incubation in the absence of CK2 and ATP (Additional file 1: Fig. S8), the band was detected after 8 h of incubation in the presence of CK2 and ATP (Fig. 5a). The intensity of the αSyn-positive band > 250 kDa of Y136A r-αSyn was significantly greater than that of WT r-αSyn after 8 h of incubation (Fig. 5b). Moreover, ThT spectroscopic assay showed an increase in fluorescence in reactions with Y136A r-αSyn in the presence of CK2 and ATP, but not in their absence, whereas no increase in fluorescence was observed with WT r-αSyn regardless of the presence or absence of CK2 and ATP within 7 days (Fig. 5c, Additional file 1: Fig. S9). Y136A r-αSyn amyloid fibrils were observed in reactions in the presence of CK2 and ATP on day 7 of incubation by TEM analysis (Fig. 5d, Additional file 1: Fig. S10). In contrast, amorphous aggregates, but not fibrils, were exclusively observed in reactions with WT r-αSyn in the presence of CK2 and ATP (Fig. 5d, Additional file 1: Fig. S10). The maximal fluorescence intensity was significantly higher in reactions with Y136A r-αSyn in the presence of CK2 and ATP than other reactions (Fig. 5e). The lag phase was significantly shorter in reactions with Y136A r-αSyn in the presence of CK2 and ATP than other reactions (Fig. 5f). These results suggest that blocking Y136 phosphorylation facilitates aggregation and amyloid fibril formation of αSyn. The insoluble aggregates > 250 kDa were detected in WT r-αSyn, but not Y136A r-αSyn, with an antibody against pY136-αSyn and in both WT and Y136A r-αSyn with an antibody against pS129-αSyn after 8 h of incubation in the presence of CK2 and ATP (Fig. 6a, b). Interestingly, the intensity of the pS129-αSyn-positive band of Y136A r-αSyn was significantly higher than that of WT r-αSyn at 8 h of incubation (Fig. 6c). Unlike WT r-αSyn, immunoreactivity with anti-pS129-αSyn antibody was more prominent in the insoluble aggregates with molecular weight > 250 kDa than 16-kDa monomer of Y136A r-αSyn at 8–40 h of incubation (Fig. 6b). The insoluble aggregates > 250 kDa were detected at relatively low levels in WT r-αSyn and Y136A r-αSyn with anti-pY125-αSyn antibody after 8 h of incubation in the presence of CK2 and ATP (Fig. 6d). The intensity of the pY125-αSyn-positive band of Y136A r-αSyn was significantly higher than that of WT r-αSyn at 8 h of incubation (Fig. 6e). Only a low level of insoluble aggregates > 250 kDa was detected in Y136A r-αSyn by anti-pY133-αSyn antibody after 32 and 40 h of incubation in the presence of CK2 and ATP, whereas no immunoreactivity was detected with anti-pY133-αSyn antibody for WT r-αSyn (Additional file 1: Fig. S11). These observations indicated that blocking of Y136 phosphorylation facilitated aggregate formation and phosphorylation at other C-terminal residues, especially S129, of r-αSyn. The results suggest that pY136 inhibits pS129 of αSyn and thereby prevents aggregate formation.

### **Exogenous oligomeric αSyn converts endogenous αSyn into insoluble aggregates with pS129 and pY136 in a prion-like manner and Y136 phosphorylation is involved in protecting against S129 phosphorylation and aggregate formation of αSyn in cultured cells**

**Fig. 7:**
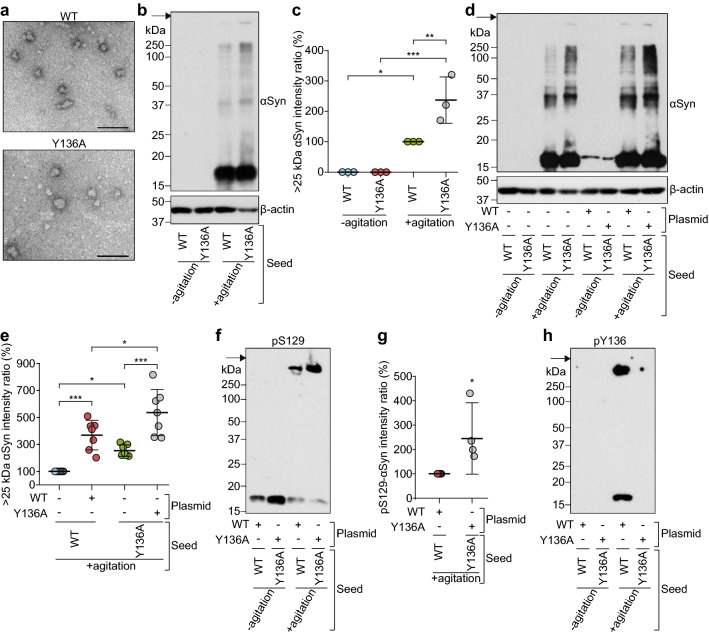
Tyr136 phosphorylation prevents aggregate formation and Ser129 phosphorylation of αSyn in cultured cells. **a** WT r-αSyn or Y136A r-αSyn subjected to agitation was examined by TEM. Bars, 100 nm. **b**–**h** WT r-αSyn or Y136A r-αSyn seed with (+ agitation) or without (− agitation) agitation was introduced into SH-SY5Y cells with (+) or without (−) pcDNA3.1 plasmid encoding WT or Y136A αSyn. The lysates from cells were analyzed by SDS-PAGE followed by immunoblotting with **b**, **d** anti-αSyn antibody Syn204, **b**, **d** anti-β-actin antibody, **f** anti-pS129-αSyn antibody D1R1R, and **h** anti-pY136-αSyn antibody. Molecular mass markers are indicated in kDa on the left side of each panel. Arrows indicate the top of the gel. Intensity ratios (%) of immunoreactive (c, e) αSyn > 25 kDa and **g** pS129-αSyn were quantified in at least three independent experiments. Data are presented as means ± standard deviation. Statistical significance was determined using **c**, **e** one-way ANOVA followed by Tukey–Kramer test and **g** 2-tailed Student’s *t* test. *P < 0.05, **P < 0.01, ***P < 0.001

We reported previously that oligomeric r-αSyn produced by agitation shows potent prion-like seeding activity in vitro by real-time quaking-induced conversion (RT-QUIC) seeding assay [29]. We confirmed the contribution of phosphorylation at S129 and Y136 to aggregate formation of αSyn in SH-SY5Y cells using oligomeric r-αSyn as seeds for prion-like propagation. WT r-αSyn and Y136A r-αSyn were converted into oligomeric forms by agitation (Fig. 7a). There were no significant differences in both forms. SDS-PAGE followed by immunoblotting analysis showed that polymers of αSyn > 25 kDa in addition to monomers accumulated in cells transfected with WT r-αSyn or Y136A r-αSyn subjected to agitation, whereas no αSyn immunoreactivity was detected in cells transfected with WT r-αSyn or Y136A r-αSyn without agitation (Fig. 7b). The levels of accumulation of αSyn > 25 kDa were significantly higher in cells transfected with agitated Y136A r-αSyn than with agitated WT r-αSyn (Fig. 7c). Furthermore, the levels of αSyn accumulation in cells transfected with agitated WT r-αSyn or Y136A r-αSyn were significantly increased by overexpression of WT αSyn or Y136A αSyn, respectively (Fig. 7d, e), indicating the prion-like seeding activity of agitated WT r-αSyn and Y136A r-αSyn in cultured cells. Detergent-insoluble aggregates > 250 kDa were detected in cells overexpressing WT αSyn or Y136A αSyn transfected with agitated, but not non-agitated, WT r-αSyn or Y136A r-αSyn using an antibody against pS129-αSyn, although 16-kDa monomer was detected in all samples (Fig. 7f). The intensity of the pS129-αSyn-positive band was significantly higher in cells transfected with agitated Y136A r-αSyn than with agitated WT r-αSyn (Fig. 7g). Immunostaining with an antibody against pY136-αSyn detected high molecular weight bands at > 250 kDa in addition to monomers only in WT αSyn-overexpressing cells transfected with agitated WT r-αSyn (Fig. 7h). Although antibodies against pY125-αSyn and pY133-αSyn also detected bands in the mass range of around 15–100 kDa in cells, the intensities of the bands were unaffected by the introduction of WT r-αSyn or Y136A r-αSyn regardless of the form of seeds (i.e., agitated or non-agitated) (Additional file 1: Fig. S12). These results suggest that oligomeric αSyn can convert native αSyn into insoluble aggregates that undergo phosphorylation of S129 and Y136, and that blocking Y136 phosphorylation facilitates aggregate formation and S129 phosphorylation of αSyn in cultured cells.

### CK2 inhibitor suppresses αSyn aggregate formation and S129 phosphorylation, and increases Y136 phosphorylation

**Fig. 8:**
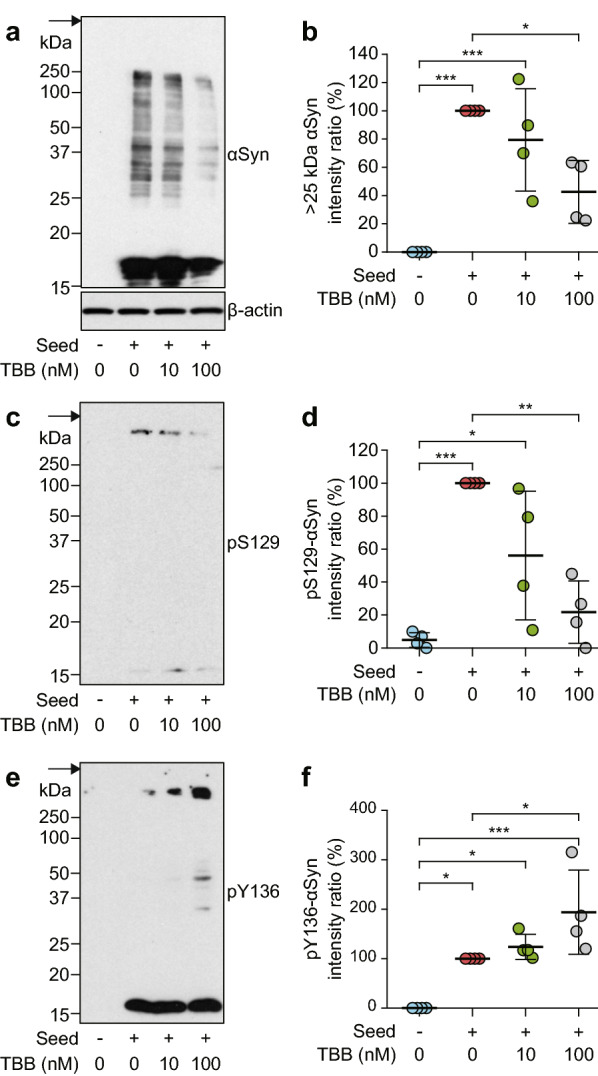
TBB suppresses αSyn aggregates through reduced Ser129 phosphorylation and increased Tyr136 phosphorylation in SH-SY5Y cells. SH-SY5Y cells treated with TBB (10 and 100 nM) or 0.05% DMSO (0) as vehicle were transfected with (+) or without (−) agitated WT r-αSyn as seed. The lysates from cells were analyzed by SDS-PAGE followed by immunoblotting with **a** anti-αSyn antibody Syn204, **a** anti-β-actin antibody, **c** anti-pS129-αSyn antibody D1R1R, and **e** anti-pY136-αSyn antibody. Molecular mass markers are indicated in kDa on the left side of each panel. Arrows indicate the top of the gel. Intensity ratios (%) of immunoreactive **b** αSyn > 25 kDa, **d** pS129-αSyn, and **f** pY136-αSyn were quantified in four independent experiments. Data are presented as means ± standard deviation. Statistical significance was determined using one-way ANOVA followed by Tukey–Kramer test. *P < 0.05, **P < 0.01, ***P < 0.001

To examine whether CK2 is involved in aggregate formation and C-terminal phosphorylation of αSyn in cells, we investigated the effects of the CK2 inhibitor, 4,5,6,7-tetrabromobenzotriazole (TBB), in SH-SY5Y cells exposed to agitated WT r-αSyn. TBB significantly reduced the levels of accumulation of αSyn > 25 kDa in a dose-dependent manner in cells transfected with agitated WT r-αSyn (Fig. 8a, b). TBB treatment also significantly reduced the intensity of the pS129-αSyn-positive band of αSyn aggregates > 250 kDa in the cells in a dose-dependent manner (Fig. 8c, d). Unexpectedly, the cells also showed an increase in the intensity of the pY136-αSyn-positive band of αSyn aggregates > 250 kDa with TBB treatment (Fig. 8e). The levels of pY136-αSyn were significantly increased by TBB in a dose-dependent manner (Fig. 8f). These results suggest that inhibition of CK2 suppresses αSyn aggregate formation by reduction of S129 phosphorylation and an unexpected increase in Y136 phosphorylation in cells.

## Discussion

The results of the present study showed that insoluble αSyn is highly phosphorylated at both Y136 and S129 in the LBD brain. In addition, pY136-αSyn, which was presumed to be oligomeric and/or ubiquitinated, was found to be constitutively expressed in the brain regardless of the presence or absence of neocortical LB. Both pY125-αSyn and pY133-αSyn were almost undetectable by immunoblotting and were detected at low levels by immunohistochemical analysis, indicating that phosphorylated αSyn is present in relatively small amounts in the brain. Moreover, Y125 and Y133 phosphorylation were unaffected by the formation of insoluble αSyn aggregates in the brain. The level of pY125 was reported to be higher in the control than LBD brain by immunoblotting, and was shown to be reduced in the aging human brain [7]. Both control subjects and LBD patients were 65–86 years old in this previous study [7], while brain tissues from patients 75–92 years of age were examined in the present study. The previous study also showed that the level of pY125 in heads from flies expressing WT human αSyn decreased with increasing incubation time at room temperature [7]. Therefore, the lack of detectable pY125 in the brain by immunoblotting was likely due to the age of the patients and the postmortem interval in the present study. Brain tissues were examined 8–12 years postmortem in this study, while the postmortem interval in the previous study was not clearly indicated. Although all tissues were stored at − 80 °C until use in this study, the postmortem interval or freeze-thawing may have been involved in the lack of detectable pY125.

Several protein kinases have been suggested to be responsible for S129 phosphorylation of αSyn, including CK1, G protein-coupled receptor kinases (GRKs), and polo-like kinases (PLKs). We also showed that CK2 phosphorylated αSyn at S129 in vitro, which was strongly correlated with αSyn aggregate formation consistent with our previous report [29]. Although CK2 has generally been classified as a serine/threonine protein kinase, several studies have demonstrated its tyrosine phosphorylation activity, suggesting that it acts as a dual-specificity kinase [3, 33, 37]. Indeed, we found that the C-terminal tyrosine residues surrounding S129, Y125 and Y136, were phosphorylated by CK2, and that tyrosine phosphorylation was exclusively found in insoluble αSyn species in vitro. In addition, insoluble αSyn aggregation was accompanied by pY136, but not pY125, as well as pS129 in cultured human cells that had taken up extracellular αSyn oligomers as seeds. These findings suggested that pY136 is related in some way to the formation and propagation of αSyn aggregates in cells. Phosphorylation at certain residues of αSyn has been shown to affect subsequent phosphorylation events in neighboring residues. Mutation of Y125 to phenylalanine (Y125F), preventing phosphorylation at this site, has been shown to decrease the levels of S129 phosphorylation by CK1 in vitro [16]. Double mutation of Y133 and Y136 to phenylalanine enhanced phosphorylation of Y125 by Lyn tyrosine kinase in vitro [22]. In our in vitro experiment, prevention of S129 phosphorylation in S129A mutant blocked Y125 and Y136 phosphorylation by CK2 with formation of αSyn aggregates, suggesting that pS129 mediates phosphorylation of Y125 and Y136 and this plays a role in αSyn aggregate formation. Prominent pS129 was seen within 1 day of incubation of WT αSyn with CK2, while pY125 and pY136 were observed after 7 and 3 days of incubation, respectively. Analysis of the kinetic parameters for phosphorylation of tyrosine-containing peptides by CK2 in vitro demonstrated that tyrosine phosphorylation is less favorable than serine/threonine phosphorylation [20]. Therefore, these results suggest that CK2 preferentially catalyzes phosphorylation of S129, which is essential for subsequent Y125 and Y136 phosphorylation in αSyn.

The substrate specificity of CK2 is determined by one or more negatively charged residues, i.e., aspartic acid (D)/glutamic acid (E), surrounding the phosphorylatable serine (S) and threonine (T) residues. The minimum consensus sequence is S/T-X-X-D/E, where X can be any amino acid. The most crucial acidic residue position for susceptibility to phosphorylation by CK2 is n + 3 followed by n + 1 [21]. In the case of αSyn, the amino acid at position n + 1 from S129 is E130 (Fig. 1a), which is predicted to mostly act as a specificity determinant for S129 phosphorylation by CK2. Little is known about the substrate specificity of CK2 for tyrosine phosphorylation. If the consensus sequence is commonly recognized by CK2 for tyrosine phosphorylation, it is possible that Y136 is the C-terminal tyrosine residue most susceptible to phosphorylation, consistent with our results, because the amino acid at positions n + 1 and n + 3 from Y136 are the acidic residues, E137 and E139, respectively, while acidic residues are present at positions n + 1 from Y125 and n + 2 from Y133, (E126 and D135, respectively) (Fig. 1a). Two members of the PLK family, PLK2 and PLK3, recognize an acidic residue similar to CK2 [27]. An in vitro study showed that PLK2, and to a lesser extent PLK3, phosphorylated S129 more efficiently than CK2 [28]. However, PLK2 knockout (KO) mice showed not over 50% decrease in pS129, while PLK3 KO had little effect on pS129 in various brain regions [4]. The remaining pS129 levels were not reduced by treatment with PLK1-3 inhibitor in PLK2 KO mice [4]. The results suggest that S129 can be phosphorylated by multiple kinases in vivo. Moreover, PLK2 KO has been reported to have no effect on pS129 in LB but not presynaptic terminals in mice [36]. Therefore, it is likely that other, non-PLK kinases, including CK2, mediate phosphorylation of S129 of αSyn aggregates in vivo. Although it remains unclear why pS129 is essential for subsequent tyrosine phosphorylation, pS129 increases the negative charge on the C-terminus by the addition of a PO_4_^2−^ group and may therefore lower the threshold for tyrosine phosphorylation by CK2. It has been reported that most of the phosphorylation sites are located in intrinsically disordered regions [12], and that phosphorylation induces folding of the intrinsically disordered protein [2, 18]. Indeed, phosphorylation of the papillomavirus E2 protein by CK2 has been reported to induce a conformational change that leads to degradation of the protein [25]. It has been also reported that pS129 increases the conformational flexibility of αSyn [24]. Therefore, it is possible that disorder-to-order conformational transitions occur in the intrinsically disordered C-terminal region of αSyn by pS129, and the conformational changes also enable phosphorylation of tyrosine residues by CK2 in addition to induction of αSyn aggregate formation. The affinity of anti-pS129-αSyn antibody could be enhanced by Y136A mutation, or the levels of pS129 may be increased by Y136A substitution itself. However, the levels of expression of αSyn and the levels of pS129 were not significantly affected by Y136A mutation in αSyn-overexpressing cells as determined by immunoblotting analysis (Additional file 1: Fig. S13). There were also no differences in the expression pattern of αSyn between WT and Y136A αSyn-overexpressing cells (Additional file 1: Fig. S13). Moreover, insoluble aggregates and amyloid fibrils of nonphosphorylated Y136A r-αSyn were not formed by incubation (Fig. 5c, Additional file 1: Fig. S7a). Therefore, it is unlikely that anti-pS129-αSyn antibody binds Y136A αSyn with different affinity than WT αSyn and that the increase in pS129 and acceleration of aggregate formation of αSyn by Y136A mutation are due to Y136A substitution itself. Although the 16-kDa monomer of WT αSyn was phosphorylated at Y136, there was no significant difference in the level of pS129 between WT and Y136A αSyn-overexpressing cells (Additional file 1: Fig. S13). These results suggest that pY136 has little effect on pS129 of αSyn monomer in non-diseased cells.

Preventing phosphorylation of Y136 by Y136A mutation facilitated aggregate formation and S129 phosphorylation of r-αSyn and αSyn in cultured cells. Y136 may also be one of the major phosphorylatable sites for CK2 in negatively charged αSyn aggregates formed with increased pS129 and to protect against further S129 phosphorylation and αSyn aggregate formation by undergoing phosphorylation instead of S129. TBB significantly inhibited S129 phosphorylation and suppressed αSyn aggregate formation in cultured cells. These results suggested that CK2 is the main protein kinase for S129 phosphorylation of αSyn and that phosphorylation of S129 by CK2 is closely related to the formation of αSyn aggregates in SH-SY5Y cells. Therefore, CK2 may be a therapeutic target for LB disease. Unexpectedly, phosphorylation of Y136 in αSyn aggregates was significantly increased by TBB in cultured cells, suggesting that CK2 plays an inhibitory role against Y136 phosphorylation in cells. As CK2 phosphorylates hundreds of physiological substrates to control various cellular processes [5], its in vitro and in vivo functions may not be consistent. CK2 has been reported to phosphorylate threonine residues of Src family tyrosine kinases, thereby resulting in reduction of their activities in vitro [38]. Therefore, the significant increase in pY136 in αSyn aggregates may be due to disinhibition of tyrosine kinases under conditions where CK2 is inhibited in cells, although the major protein kinases for phosphorylation of Y136 have yet to be elucidated, and may contribute to reduction in pS129 and confer protection against αSyn aggregate formation.

## Conclusions

The findings of the present study provide the first evidence that CK2 phosphorylates Y136 in αSyn aggregates by mediating S129 phosphorylation as a dual-specificity kinase and that pY136 has a protective effect against αSyn aggregation. Although the primary kinase for pY136 in vivo is not yet clear, this study suggested that CK2 is a candidate kinase responsible for pY136 and that it participates in regulating αSyn aggregate formation. Further studies are necessary to elucidate the potential roles of the interactions between CK2 and αSyn C-terminal phosphorylation, their involvement in the pathogenesis of LB diseases, and their potential for the development of novel therapeutic strategies.

## Supplementary Information

**Additional file1.** Supplementary fgures.

## Data Availability

All data generated or analyzed during this study are included in this published article and its supplementary information files.

## Abbreviations

AD: Alzheimer’s disease
CK1: Casein kinase 1
CK2: Casein kinase 2
CJD: Creutzfeldt–Jakob disease
LB: Lewy body
LBD: Lewy body dementia
LC–MS/MS: Liquid chromatography-ion trap mass spectrometry
MS: Mass spectrometry
PD: Parkinson’s disease
TBB: 4,5,6,7-Tetrabromobenzotriazole
TEM: Transmission electron microscopy
ThT: Thioflavin T
αSyn: α-Synuclein
WT: Wild-type
