# Supplementary material for: Tyrosine 136 phosphorylation of α-synuclein aggregates in the Lewy body dementia brain: involvement of serine 129 phosphorylation by casein kinase 2
Source: Acta Neuropathol Commun. 2021 Nov 12;9:182. doi: 10.1186/s40478-021-01281-9 (PMC8590312; doi:10.1186/s40478-021-01281-9)
Supplement: Supplementary file 1 — Additional file1. Supplementary fgures. [file 40478_2021_1281_MOESM1_ESM.doc]

**Additional file 1**

**Tyrosine 136 phosphorylation of** **α-synuclein aggregates in the Lewy body dementia brain: involvement of serine 129 phosphorylation by casein kinase 2**

Kazunori Sano^1*^, Yasushi Iwasaki^2^, Yuta Yamashita^1^, Keiichi Irie^1^, Masato Hosokawa^3^, Katsuya Satoh^4^, Kenichi Mishima^1^

^1^Department of Physiology and Pharmacology, Faculty of Pharmaceutical Sciences, Fukuoka University, Fukuoka 814-0180, Japan

^2^Department of Neuropathology, Institute for Medical Science of Aging, Aichi Medical University, Aichi 480-1195, Japan

^3^Department of Immunological and Molecular Pharmacology, Faculty of Pharmaceutical Sciences, Fukuoka University, Fukuoka 814-0180, Japan

^4^Department of Health Sciences, Unit of Medical and Dental Sciences, Nagasaki University Graduate School of Biomedical Sciences, Nagasaki 852-8523, Japan

^*^Corresponding author: Kazunori Sano

Department of Physiology and Pharmacology, Faculty of Pharmaceutical Sciences, Fukuoka University, 8-19-1 Nanakuma, Jonan-ku, Fukuoka 814-0180, Japan. Tel.: +81-92-871-6631, Fax: +81-92-863-0389, E-mail: [ksano@fukuoka-u.ac.jp](mailto:ksano@fukuoka-u.ac.jp)


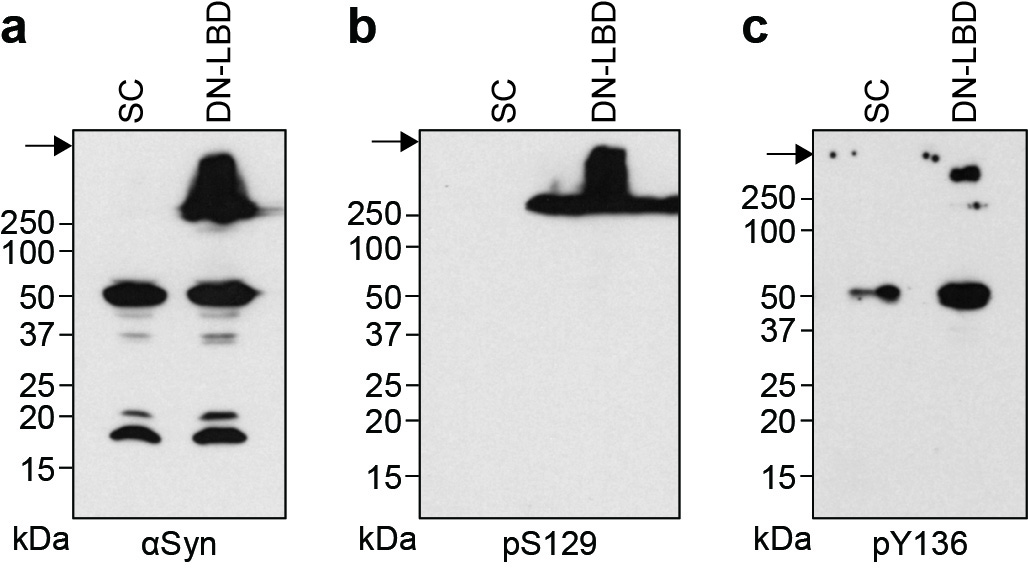


**Figure S1 The formation of insoluble αSyn aggregates was not observed in the brain of a schizophrenia patient.** (a – c) Brain lysates from patients with DN-LBD (cases #2) and schizophrenia (SC, 37 years old) were analyzed by SDS-PAGE followed by immunoblotting with (a) anti-αSyn antibody D119, (b) anti-pS129-αSyn antibody and (c) anti-pY136-αSyn antibody. Molecular mass markers are indicated in kDa on the left side of each panel. Arrows indicate the top of the gel.


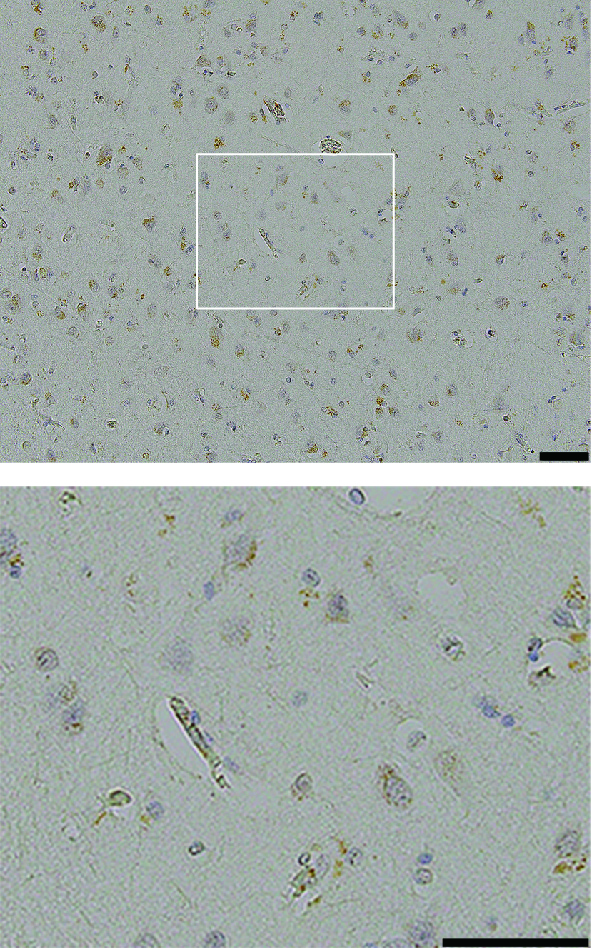


**Figure S2 Deposits were not detected in the brain of a non-DLB patient by immunohistochemical analysis using anti-pS129-αSyn antibody.** Immunohistochemical staining using antibody against pS129-αSyn in the frontal cortex from non-DLB case (breast cancer patient, 91 years old). Regions surrounded by white rectangles in the upper panels are magnified and shown in the lower panels. Scale bars, 50 μm.


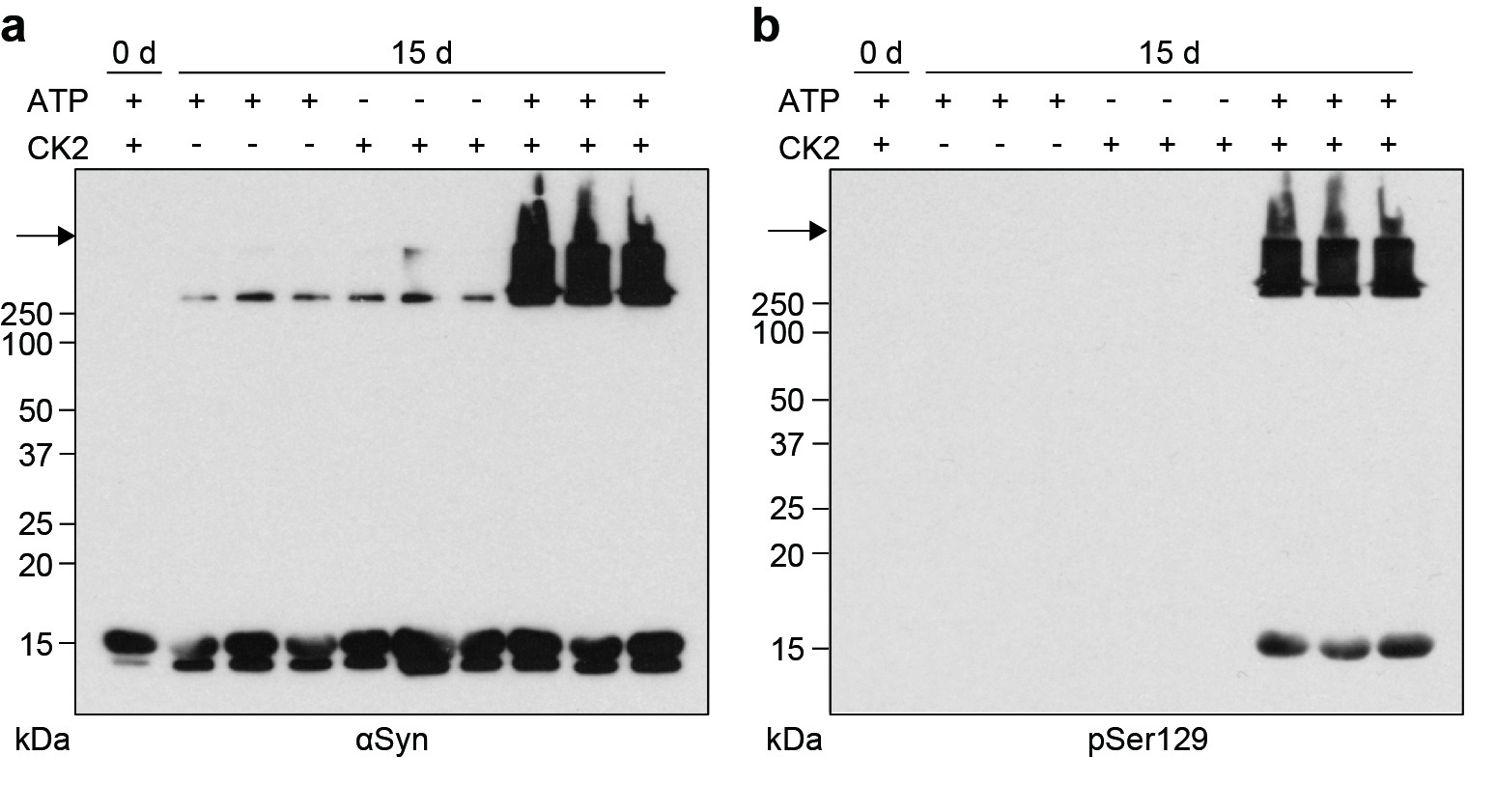


**Figure S3 Ser129 phosphorylation by CK2 accelerates the formation of insoluble r-αSyn aggregates.** WT r-αSyn was incubated in the presence (+) or absence (−) of CK2 or ATP. After 0 or 15 days of incubation, the samples were analyzed by SDS-PAGE followed by immunoblotting with (a) anti-αSyn antibody D119 and (b) anti-pSer129-αSyn antibody ab51253. Molecular mass markers are indicated in kDa on the left side of each panel. Arrows indicate the top of the gel.


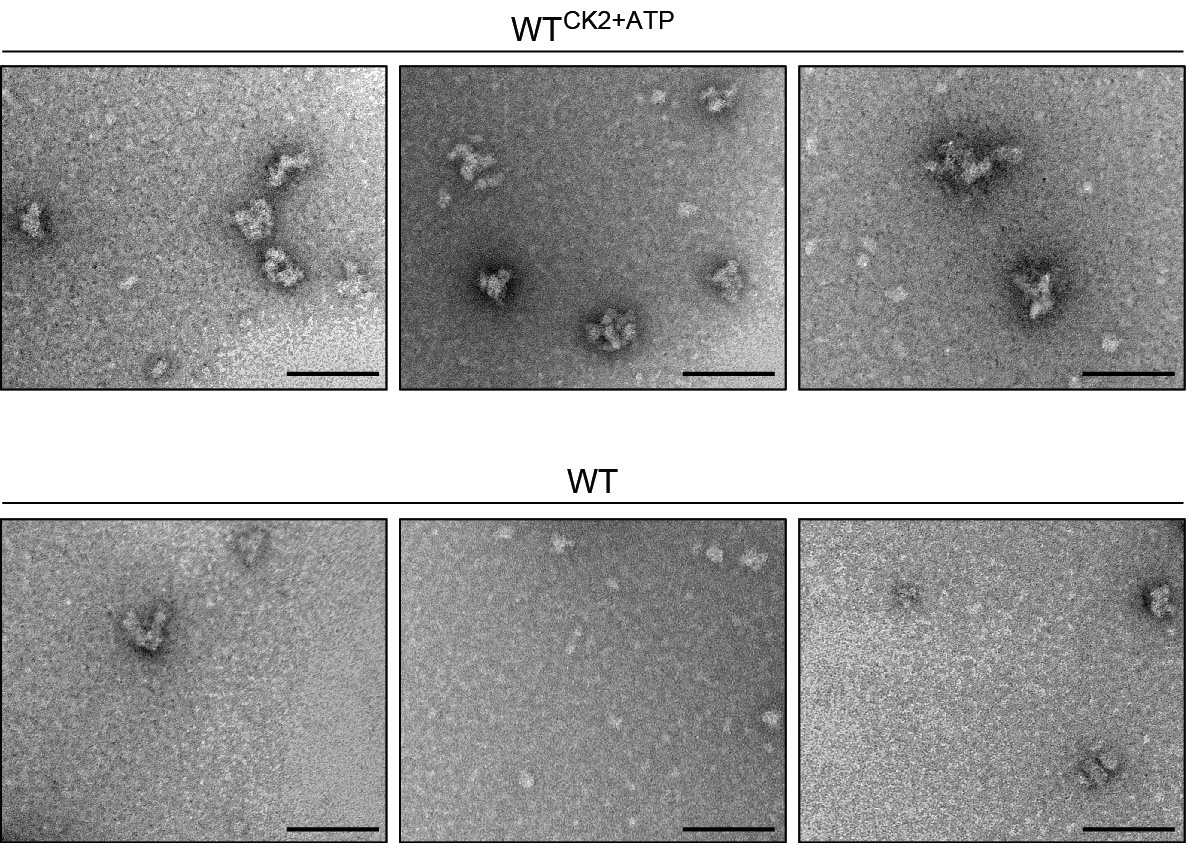


**Figure S4 Ser129 phosphorylation by CK2 accelerates the formation of amorphous aggregates of r-αSyn.** WT r-αSyn after 15 days of incubation in the presence (WT^CK2+ATP^) or absence (WT) of CK2 and ATP was examined by TEM. Bars, 100 nm.


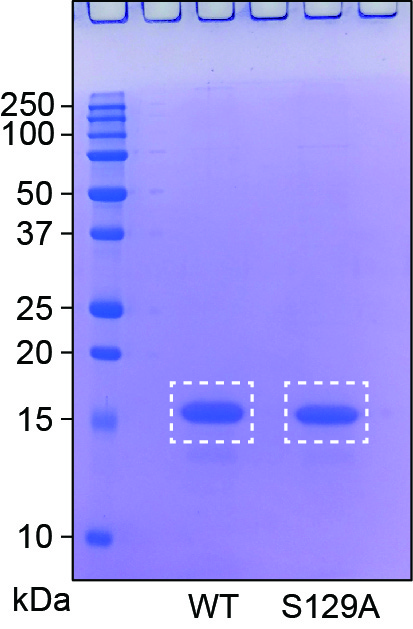


**Figure S5 Coomassie Brilliant Blue stained SDS-PAGE with r-αSyn incubated with CK2 and ATP.** After WT r-αSyn or S129A r-αSyn was incubated in the presence of CK2 and ATP for 7days, the samples were subjected to SDS-PAGE followed by Coomassie Brilliant Blue staining. A protein band corresponding to a molecular weight of around ~16kDa surrounded by white dashed line was excised and digested with trypsin. The digest was analyzed with LC-MS/MS. Molecular mass markers are indicated in kDa on the left side of each panel.


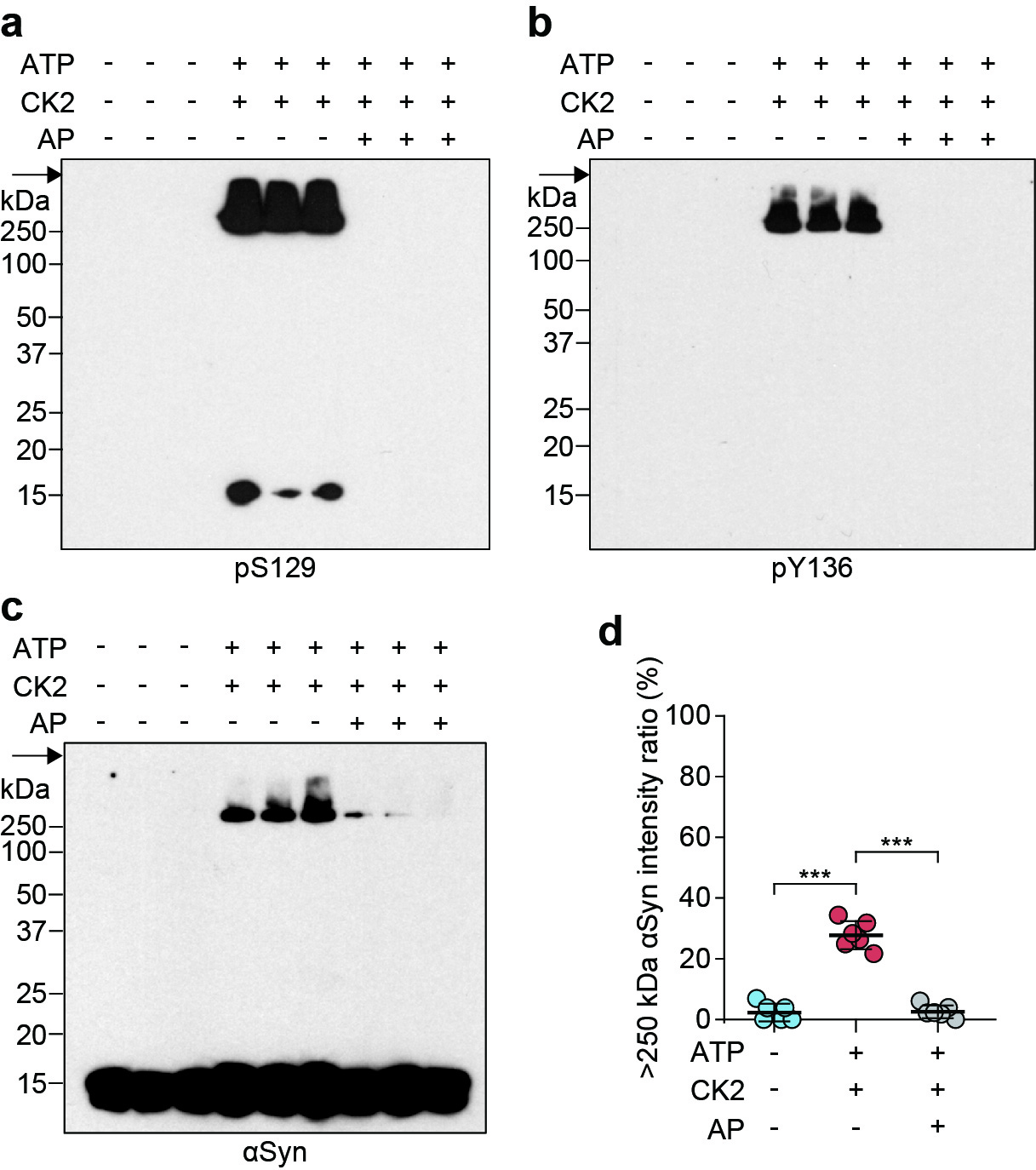


**Figure S6 pS129 and pY136 of r-αSyn and the formation of insoluble aggregates were inhibited by alkaline phosphatase.** WT r-αSyn after 3 days of incubation in the presence (+) or absence (−) of ATP, CK2 or 5 U/μl alkaline phosphatase (AP, 2250A; Takara Bio) was analyzed by SDS-PAGE followed by immunoblotting with (a) anti-pS129-αSyn antibody ab51253, (b) anti-pY136-αSyn antibody and (c) anti-αSyn antibody D119. Molecular mass markers are indicated in kDa on the left side of each panel. Arrows indicate the top of the gel. (d) Intensity ratios (%) of immunoreactive > 250 kDa αSyn after 3 days of incubation were quantified in six independent experiments. Data are presented as means ± standard deviation. Statistical significance was determined using one-way ANOVA followed by Tukey–Kramer test. ***P < 0.001.


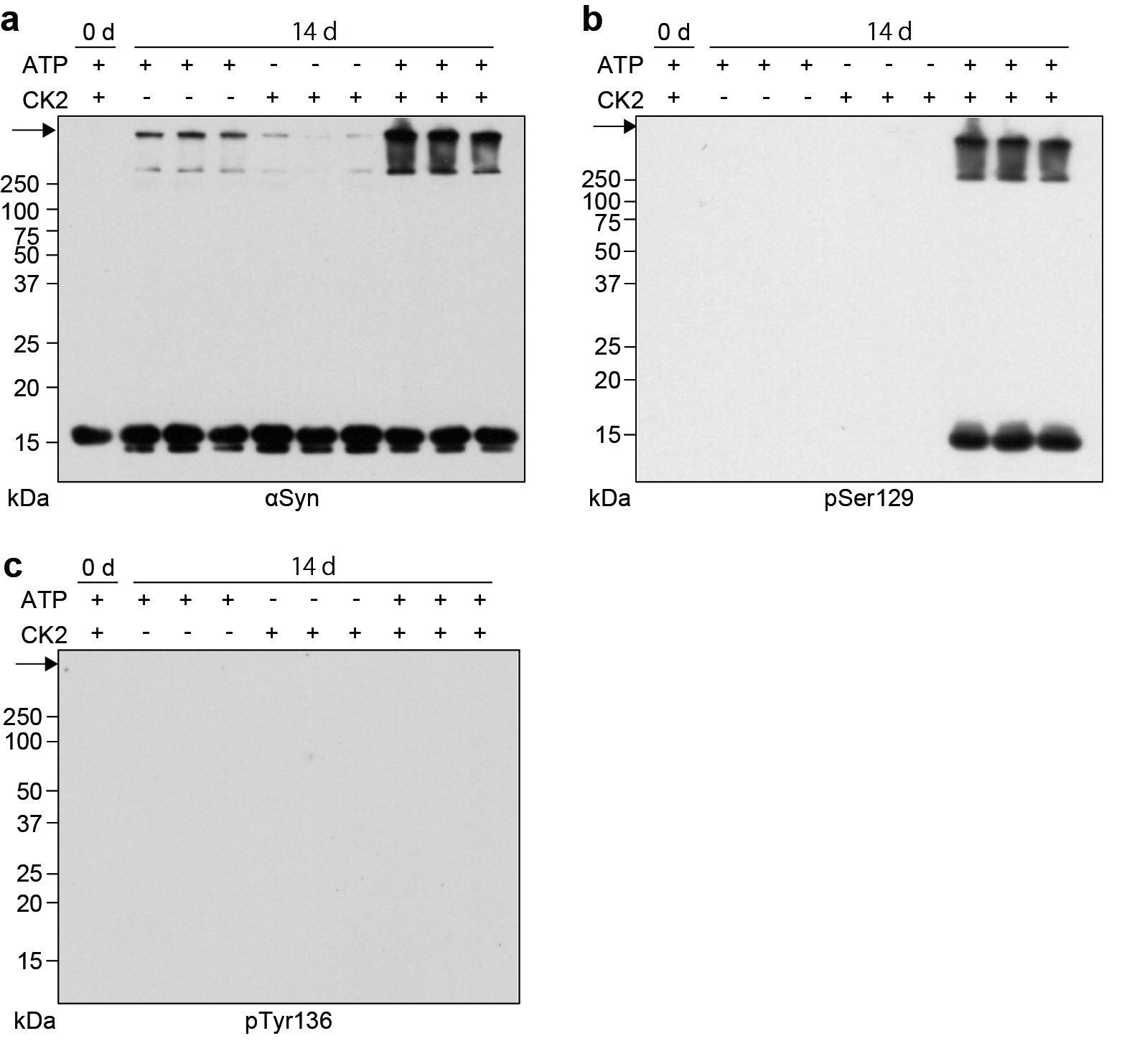


**Figure S7 Y136A r-αSyn is phosphorylated at Ser129 by CK2 and formed insoluble aggregates.** Y136A r-αSyn after 0 or 14 days of incubation in the presence (+) or absence (−) of CK2 or ATP was analyzed by SDS-PAGE followed by immunoblotting with (a) anti-αSyn antibody D119, (b) anti-pS129-αSyn antibody ab51253 and (c) anti-pY136-αSyn antibody. Molecular mass markers are indicated in kDa on the left side of each panel. Arrows indicate the top of the gel.


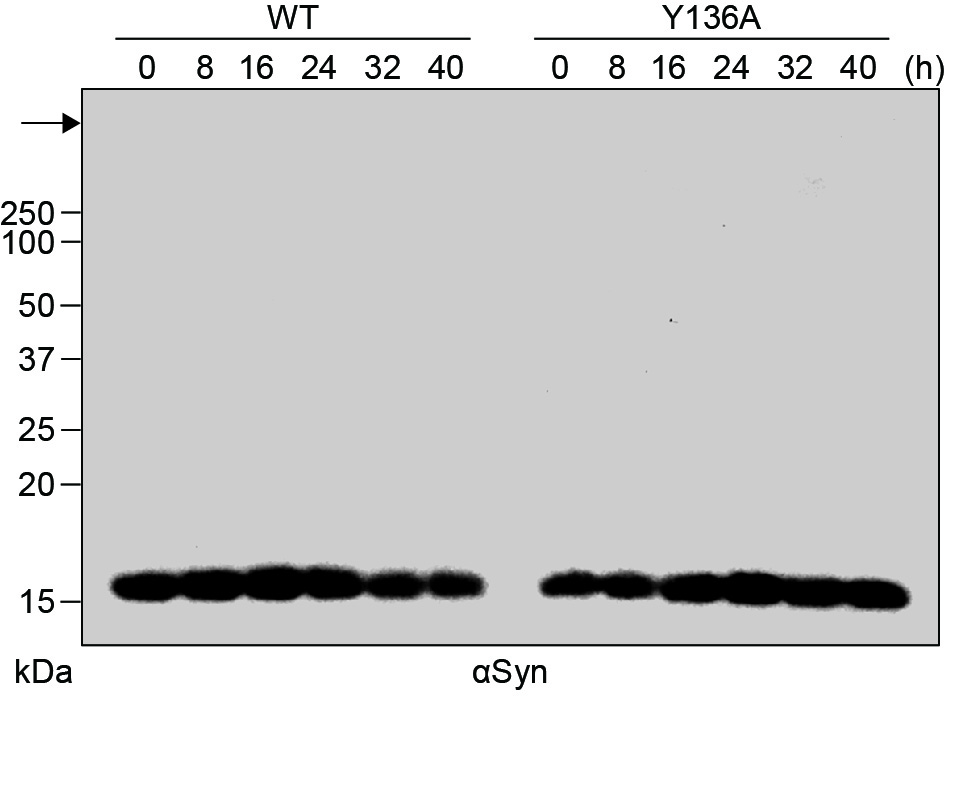


**Figure S8 Insoluble aggregates of WT r-αSyn and Y136A r-αSyn was not observed within 40 h of incubation in the absence of CK2 and ATP.** WT r-αSyn or Y136A r-αSyn after 0 – 40 hours of incubation in the absence of CK2 and ATP was analyzed by SDS-PAGE followed by immunoblotting with anti-αSyn antibody D119. Molecular mass markers are indicated in kDa on the left side of each panel. Arrows indicate the top of the gel.

**
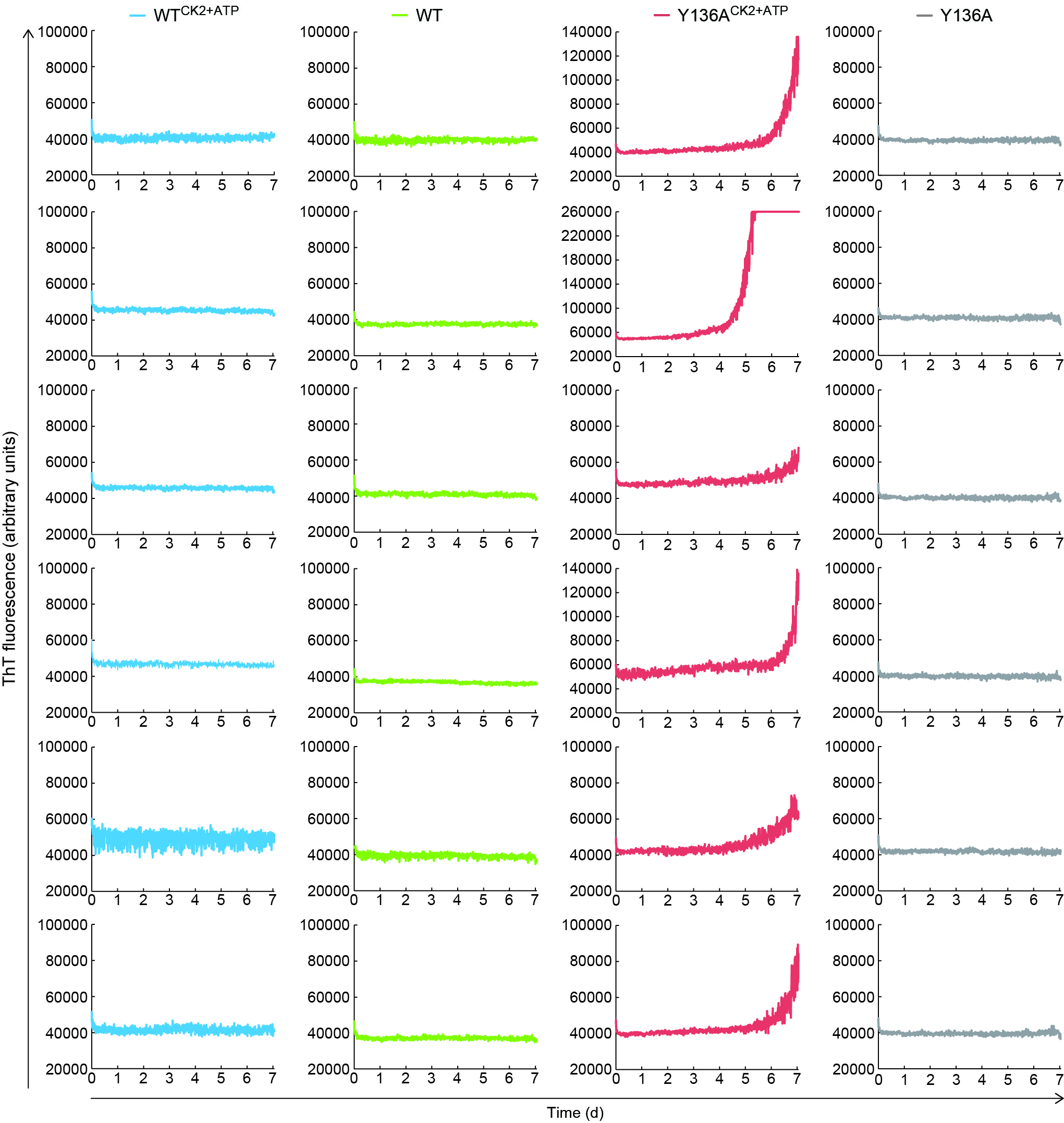
**

**Figure S9 ThT assay showed an increase in fluorescence in reactions with Y136A r-αSyn in the presence of CK2 and ATP.** ThT assays were performed in reactions with WT r-αSyn in the presence (WT^CK2+ATP^) or absence (WT) of CK2 and ATP or in reactions with Y136A r-αSyn in the presence (Y136A^CK2+ATP^) or absence (Y136A) of CK2 and ATP. All reactions were performed in six replicates. The colored curves represent the kinetics of the ThT fluorescence from an individual reaction.


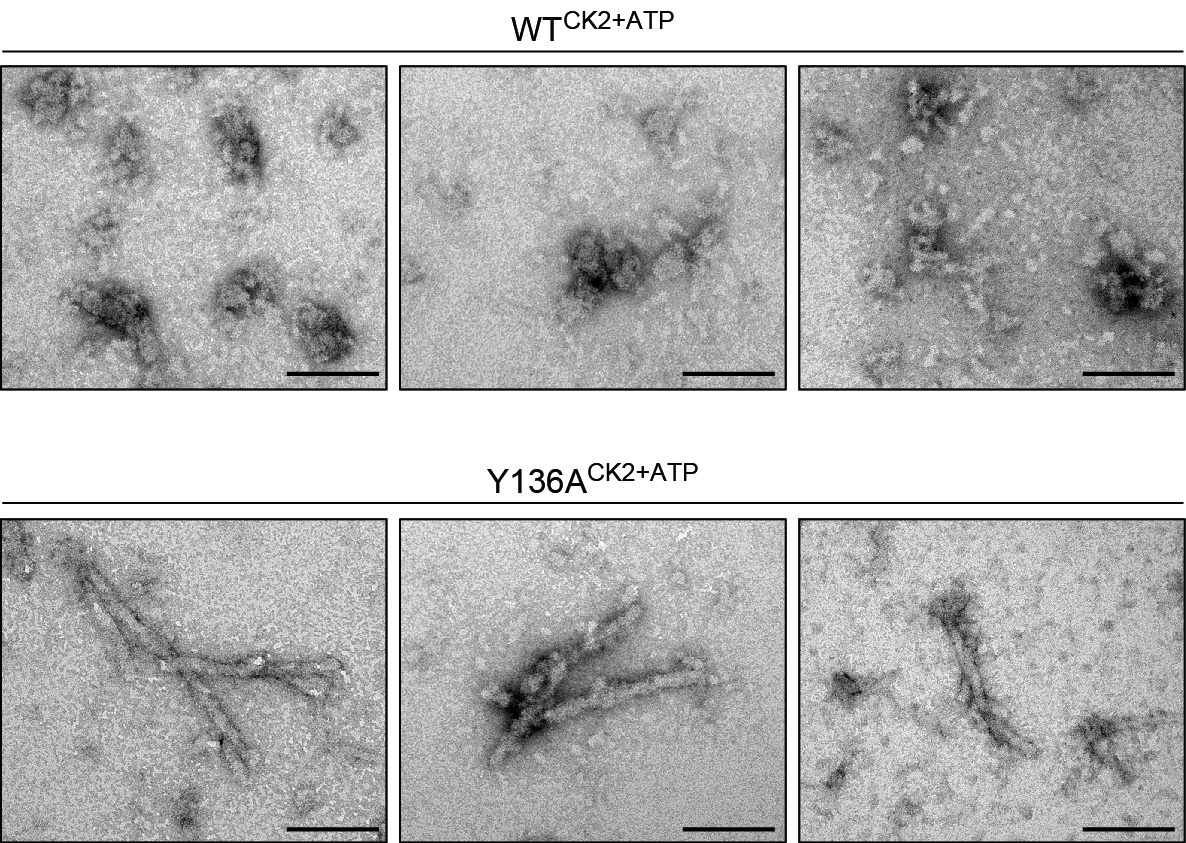


**Figure S10 Amyloid fibrils were observed in reactions with Y136A r-αSyn, but not WT r-αSyn, in the presence of CK2 and ATP of ThT assay.** The end products from reactions with WT r-αSyn (WT^CK2+ATP^) or Y136A r-αSyn (Y136A^CK2+ATP^) in the presence of CK2 and ATP in ThT assay were examined by TEM. Bars, 100 nm.


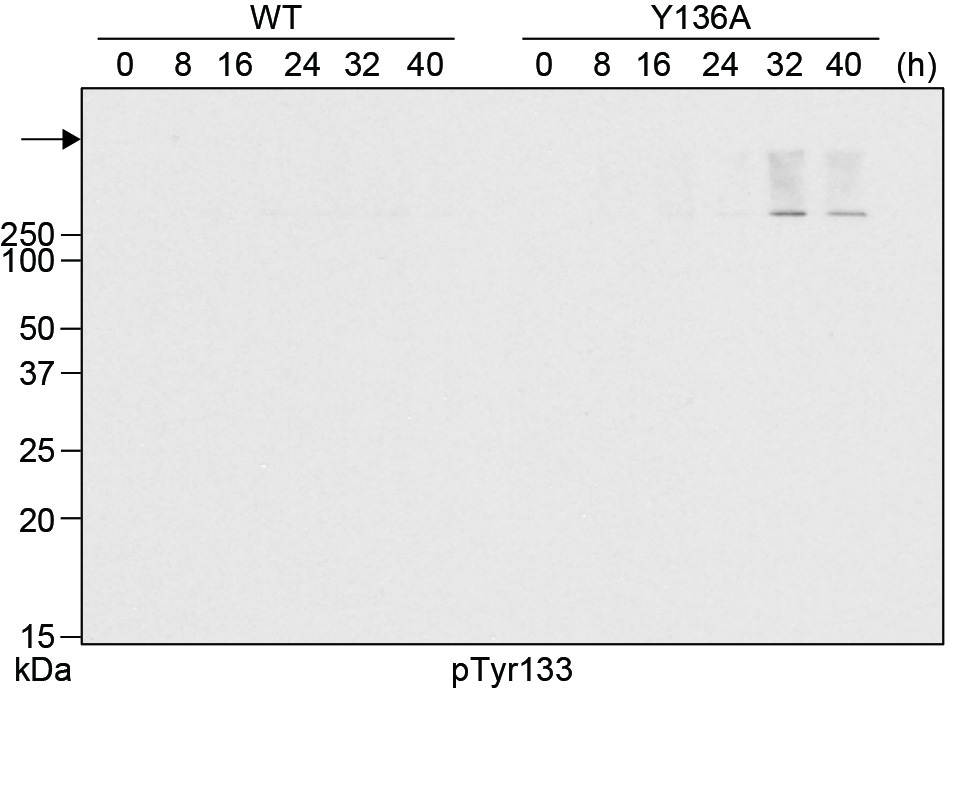


**Figure S11 Only a few insoluble aggregates phosphorylated at Tyr133 were detected in Y136A r-αSyn but not WT r-αSyn after incubation in the presence of CK2 and ATP.** WT r-αSyn or Y136A r-αSyn after 0 – 40 hours of incubation in the presence of CK2 and ATP was analyzed by SDS-PAGE followed by immunoblotting with anti-pY133-αSyn antibody. Molecular mass markers are indicated in kDa on the left side of each panel. Arrows indicate the top of the gel.


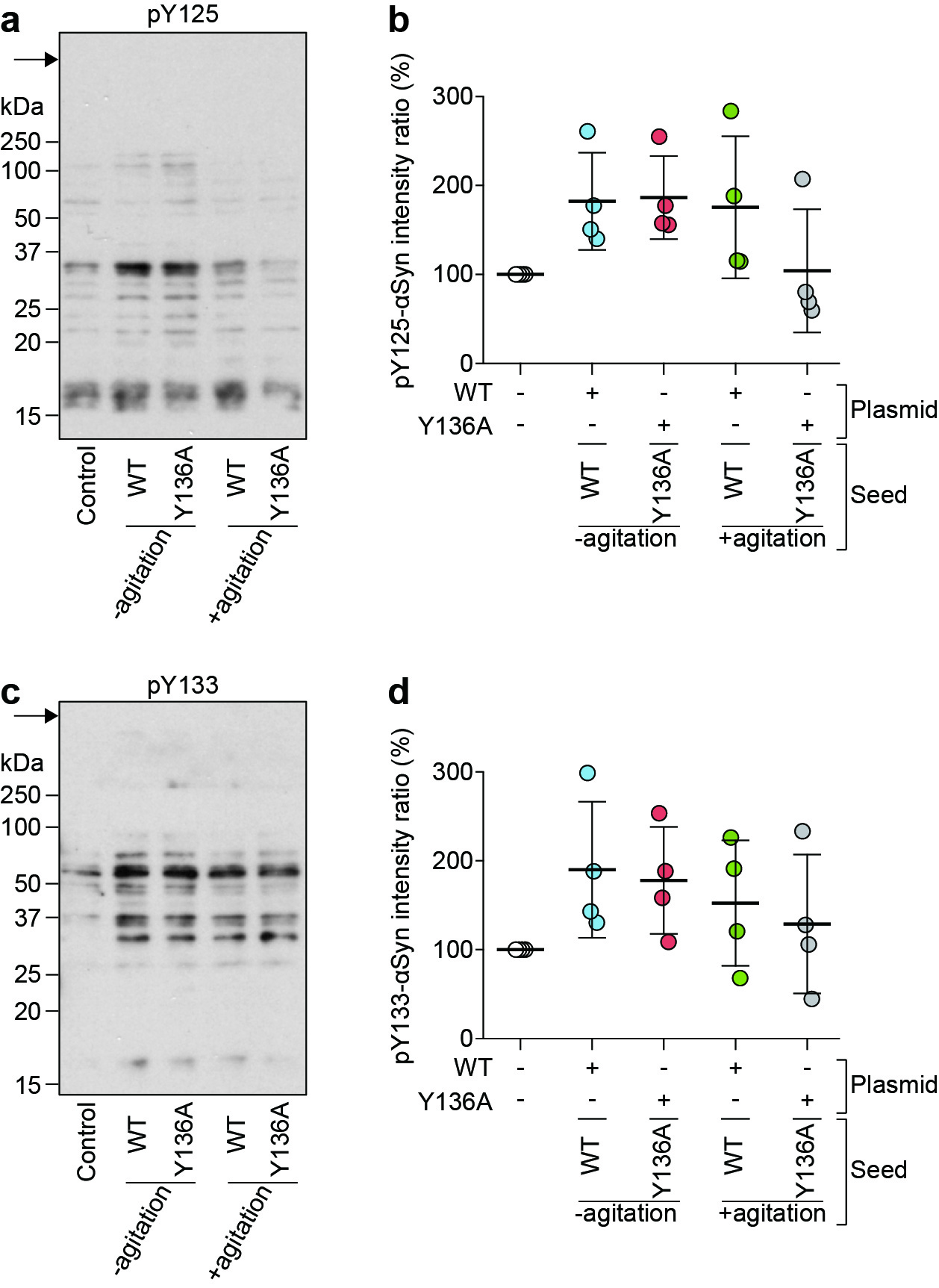


**Figure S12 No significant changes in Tyr125 and Tyr133 phosphorylation of αSyn were observed in cultured cells transfected with r-αSyn seeds.** WT r-αSyn or Y136A r-αSyn seed with (+agitation) or without (−agitation) agitation was introduced into SH-SY5Y cells with (+) or without (−) pcDNA3.1 plasmid encoding WT or Y136A αSyn. The lysates from cells were analyzed by SDS-PAGE followed by immunoblotting with (a) anti-pY125-αSyn antibody and (c) anti-pY133-αSyn antibody. Molecular mass markers are indicated in kDa on the left side of each panel. Arrows indicate the top of the gel. Intensity ratios (%) of immunoreactive (b) pY125-αSyn and (d) pY133-αSyn were quantified in four independent experiments. Data are presented as means ± standard deviation. Statistical significance was determined using one-way ANOVA followed by Tukey–Kramer test.

**
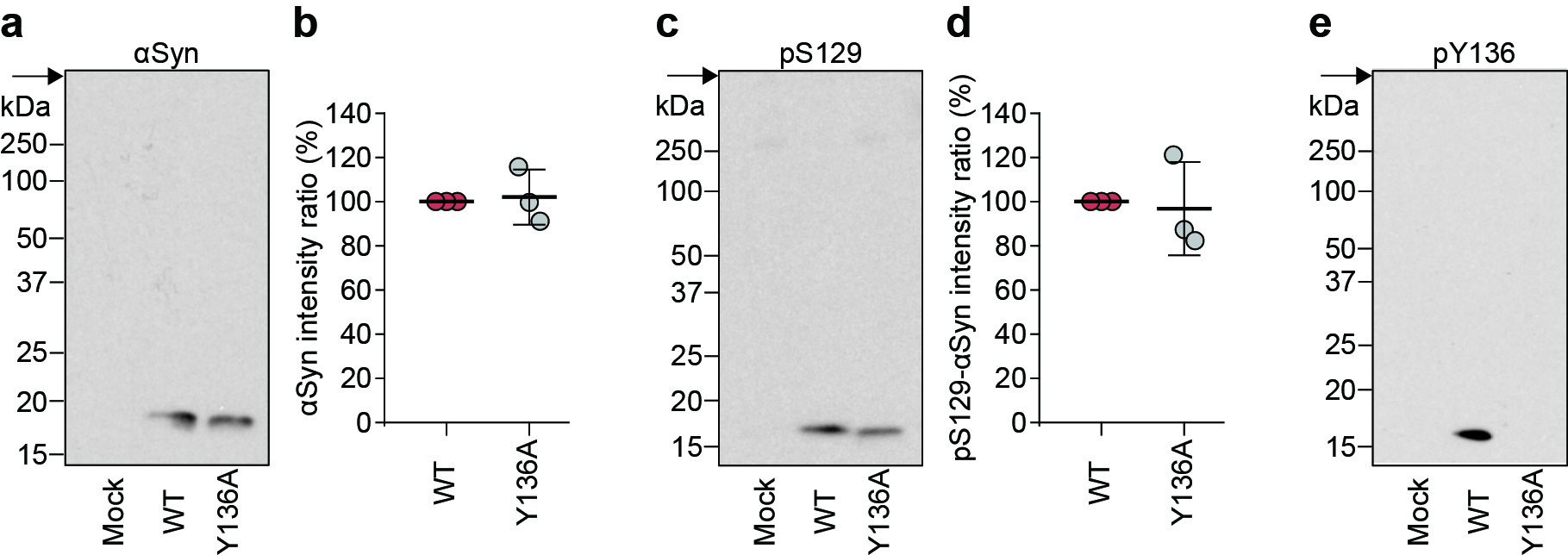
**

**Figure S13 There were no differences in the levels of expression of αSyn and the level of pS129** **between WT and Y136A αSyn-overexpressing cells.** pcDNA3.1 plasmid encoding WT or Y136A αSyn, or the empty plasmid (Mock) was transfected into SH-SY5Y cells. The lysates from cells were analyzed by SDS-PAGE followed by immunoblotting with (a) anti-αSyn antibody Syn204, (c) anti-pS129-αSyn antibody D1R1R and (e) anti-pY136-αSyn antibody. Molecular mass markers are indicated in kDa on the left side of each panel. Arrows indicate the top of the gel. Intensity ratios (%) of immunoreactive (b) αSyn and (d) pS129-αSyn were quantified in three independent experiments. Data are presented as means ± standard deviation. Statistical significance was determined using the 2-tailed Student’s *t* test.
